# Restoring NK Cell Cytotoxicity Post‐Cryopreservation via Synthetic Cells

**DOI:** 10.1002/advs.202505731

**Published:** 2025-09-18

**Authors:** Xiangda Zhou, Sijia Zhang, Wenjuan Yang, Susanne Gonder, Zeinab Sadjadi, Nils Piernitzki, Alina Moter, Shulagna Sharma, Anne Largeot, Nadja Küchler, Lea Kaschek, Gertrud Schäfer, Eva C. Schwarz, Hermann Eichler, Evelyn Ullrich, Heiko Rieger, Oskar Staufer, Jérôme Paggetti, Etienne Moussay, Markus Hoth, Bin Qu

**Affiliations:** ^1^ Department of Biophysics Center for Integrative Physiology and Molecular Medicine (CIPMM) School of Medicine Saarland University 66421 Homburg Germany; ^2^ Reproductive Medicine Center Second Affiliated Hospital of Naval Medical University Shanghai 200003 China; ^3^ Tumor Stroma Interactions Department of Cancer Research Luxembourg Institute of Health Luxembourg L‐1445 Luxembourg; ^4^ Department of Theoretical Physics and Center for Biophysics Saarland University 66123 Saarbrücken Germany; ^5^ INM‐Leibniz Institute for New Materials Campus D2 2 66123 Saarbrücken Germany; ^6^ Goethe University Frankfurt Department of Pediatrics Experimental Immunology & Cell Therapy 60323 Frankfurt (Main) Germany; ^7^ Goethe University Frankfurt Frankfurt Cancer Institute 60596 Frankfurt (Main) Germany; ^8^ Institute for Clinical Hemostaseology and Transfusion Medicine School of Medicine Saarland University 66421 Homburg Germany; ^9^ German Cancer Consortium (DKTK) partner site Frankfurt/Mainz 60590 Frankfurt (Main) Germany; ^10^ Helmholtz Institute for Pharmaceutical Research Saarland Helmholtz Center for Infection Research Campus E8 1 66123 Saarbrücken Germany; ^11^ Saarland University Center for Biophysics Campus Saarland 66123 Saarbrücken Germany; ^12^ Max Planck Bristol Centre for Minimal Biology Cantock's Close Bristol BS8 1TS UK; ^13^ Department of Biomedical Sciences Institute for Health Research and Education Osnabrück University 49076 Osnabrück Germany

**Keywords:** cryopreservation, cytotoxicity, NK cells, synthetic cells, T cells, IL‐2, 3D

## Abstract

Natural killer (NK) cells are critical components of the first‐line immune defense, responsible for eliminating tumorigenic cells. NK cell‐based adoptive immunotherapy has gained increasing attention; however, cryopreservation, a standard technique for NK cell storage, significantly impairs NK cell cytotoxicity, particularly in physiological 3D environments. Here, we demonstrate that short‐term co‐culture with effector T cells markedly enhances NK cell motility and killing functionality. Notably, a brief 1‐day co‐culture is sufficient to restore cryopreservation‐impaired NK cell functionality in 3D environments. This enhancement requires direct contact between T cells and NK cells, which facilitates localized high concentrations of IL‐2 at the cell contact sites. To develop a controled, donor‐independent solution, we demonstrate that synthetic T cells with surface‐bound IL‐2 exhibit superior efficiency in revitalizing cryopreserved NK cells. These findings uncover a previously unrecognized role for physical contact‐mediated local IL‐2 signaling and provide an efficient, cost‐effective, and tunable strategy to rescue NK cell functionality post‐cryopreservation, paving the way for more scalable, potent, and clinically viable NK cell‐based immunotherapies.

## Graphical abstract

In this study, it is shown that 1‐day co‐culture with activated T cells or IL‐2‐presenting synthetic cells significantly enhances NK cell motility and cytotoxicity in 2D and 3D. Notably, synthetic cells restore the impaired cytotoxic function of cryopreserved NK and CAR‐NK cells in 3D. This approach offers a potent and clinically relevant strategy to improve off‐the‐shelf NK cell‐based therapies. (The graphical abstract is created using BioRender).

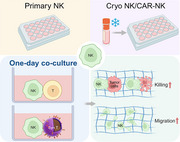

## 1 Introduction

Natural killer (NK) cells are a vital component of the innate immune system, playing a crucial role in eliminating pathogen‐infected and tumorigenic cells.^[^ ^1^ ^]^ To effectively identify and eliminate their targets, NK cells require proper mobility, enabling them to patrol tissues and locate potential threats.^[^ ^2^, ^3^ ^]^ Once an NK cell recognizes a target cell, it establishes a close interaction known as the immunological synapse (IS).^[^ ^4^ ^]^ At this interface, cytotoxic protein‐containing lytic granules (LGs) accumulate and are subsequently released into the IS cleft.^[^ ^5^, ^6^ ^]^ Among cytotoxic proteins, the pore‐forming protein perforin and serine proteases known as granzymes are key effector molecules that work synergistically to induce target cell destruction.^[^ ^7^, ^8^ ^]^ NK cells recognize and eliminate target cells through two primary mechanisms: natural cytotoxicity and antibody‐dependent cell‐mediated cytotoxicity (ADCC).^[^ ^9^ ^]^ In natural cytotoxicity, NK cells target cells that exhibit low expression of inhibitory receptor ligands (e.g., MHC I molecules) or high expression of activating receptor ligands.^[^ ^10^ ^]^ In ADCC, NK cells are recruited to antibody‐coated target cells, where the antibody binds to its specific antigen on the target cell, while its Fc region interacts with Fc receptors on NK cells.^[^ ^11^, ^12^ ^]^

NK cells have emerged as promising candidates for adoptive immunotherapy due to their potent anti‐tumor activity and relatively lower cytotoxic effects on the central neural system.^[^ ^13^, ^14^, ^15^ ^]^ However, to meet the demands of clinical applications, particularly for the development of readily available, off‐the‐shelf NK cell‐based therapies, establishing a reliable and efficient method for preserving large quantities of NK cells is crucial.^[^ ^16^ ^]^ Cryopreservation remains the most widely used method for long‐term storage of cells, relying on cryoprotectants to mitigate intracellular damage caused by ice crystal formation.^[^ ^17^ ^]^ Despite advancements in cryopreservation techniques that maintain high cell viability,^[^ ^17^ ^]^ studies indicate that the process significantly impairs NK cell migration and cytotoxic function in 3D environments.^[^ ^18^ ^]^ This functional decline presents a major challenge in scaling up NK cell production for immunotherapy, as their full efficacy is essential for therapeutic success.

In this study, we demonstrate that co‐culturing NK cells with T cells provides a rapid and highly efficient approach to enhancing NK cell motility and killing function in both 2D and 3D environments. Notably, this strategy also restores the impaired killing functionality of cryopreserved NK cells in 3D settings. We further identified that both direct NK‐T cell physical contact and IL‐2 signaling are necessary for this enhancement. To optimize this effect in a more controllable manner and minimize potential adverse side effects, we also used synthetic T cells engineered with surface‐bound IL‐2, which exhibited superior efficiency in enhancing NK cell cytotoxic functionality. Our findings offer a promising and physiological relevant strategy for rapidly boosting NK cell potency, providing a valuable solution to overcome the functional limitations of NK cells associated with cryopreservation.

## 2 Results

### 2.1 NK Cell Killing Kinetics is Substantially Accelerated by Co‐Culturing with Activated T Cells

**Figure 1:**
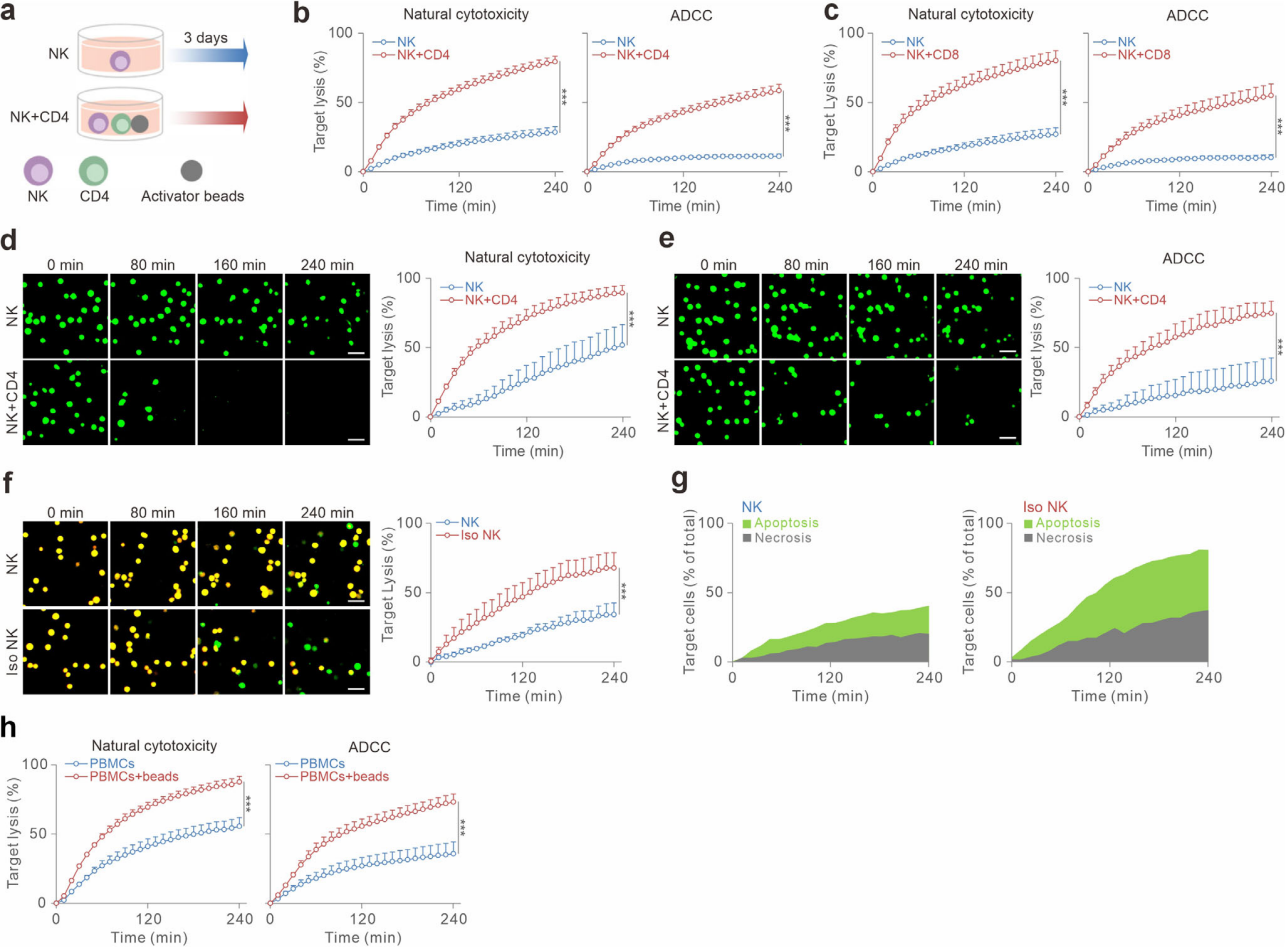
T cell co‐culture enhances NK cell cytotoxicity in 2D and 3D environments. a) Schematic of the NK‐T cell co‐culture workflow. b,c) Killing kinetics of NK cells in 2D settings measured using a plate reader‐based 2D real‐time killing assay. Primary human NK cells were cultured alone or co‐cultured with bead‐stimulated CD4⁺ (b) or CD8⁺ (c) T cells at an NK:T ratio of 1:2 for 3 days. Cytotoxicity was assessed against K562 (natural cytotoxicity) or Raji (ADCC) cells with an effector‐to‐target ratio (E:T) of 2.5:1. Fluorescence was measured every 10 min for 4 h at 37 °C. Data are presented as mean ± SEM (biological replicates: n = 20 for b, n = 4 for c). d,e) NK cell killing efficiency in 2D settings assessed by live‐cell imaging. Target cells (K562 in d, Raji in e) were loaded with calcein and seeded with NK cells (E:T = 2.5:1). Killing events were visualized every 10 min for 4 h at 37 °C using high‐content imaging (ImageXpress, 20× objective). Scale bars: 40 µm. Time‐lapse images from one representative donor are shown. Quantifications are presented as mean ± SEM (biological replicates: n = 4). f) Killing dynamics of isolated NK cells from NK‐T co‐culture. NK cells were isolated with CD56 magnetic beads from NK‐T co‐culture (Iso NK) or NK cultured alone (NK). Cytotoxicity against K562‐pCasper cells (E:T = 2.5:1) was assessed via live‐cell imaging every 10 min for 4 h at 37 °C using high‐content imaging (ImageXpress, 20× objective). Images from one representative donor are shown. Quantifications are presented as mean ± SEM (biological replicates: n = 4). Scale bars: 40 µm. g) NK cell‐mediated killing mode. Apoptosis and necrosis events were quantified from f as described in the Methods. Quantification from a representative donor is shown. h) Activation of T cells in PBMCs enhances NK cell‐mediated cytotoxicity. PBMCs were stimulated with beads for 3 days. K562 cells were used as target cells (PBMCs:K562 = 20:1, corresponding to an effective NK:K562 ratio of ≈1:1 as NK cells make up ≈5% of PBMCs on average^[^ ^57^ ^]^). 2D killing kinetics was determined by a plate reader‐based real‐time killing assay every 10 min for 4 h at 37 °C. Results are shown as mean ± SEM (biological replicates: n = 4). Statistical analysis was conducted via a two‐way ANOVA with multiple comparisons for b–f, h.

To enhance NK killing capacity, the cytokine IL‐2 is widely used.^[^ ^19^, ^20^ ^]^ However, since this cytokine requires a few days to significantly boost NK functionality, this approach may be too slow for off‐the‐shelf clinical applications. Seeking a faster alternative, we turned our attention to T cells, as they are the primary source of IL‐2, particularly CD4^+^ T cells.^[^ ^19^, ^20^ ^]^ To investigate whether T cells can enhance NK cell killing function, we co‐cultured autologous primary human CD4^+^ T cells with NK cells and stimulated the T cells for three days using anti‐CD3/anti‐CD28 antibody‐coated beads (hereafter referred to as beads) (**Figure** **1**a). To assess NK cell killing efficiency, we used K562 cells as target cells, which lack MHC class I molecules and can be directly recognized by NK cells, a process known as natural cytotoxicity. Using a time‐resolved real‐time killing assay,^[^ ^21^ ^]^ we found that NK cells co‐cultured with CD4^+^ T cells exhibited substantially enhanced killing activity compared to NK cells alone (Figure 1b, left panel). Concomitantly, antibody‐dependent cell‐mediated cytotoxicity (ADCC) was markedly increased by co‐culture with activated CD4^+^ T cells, as demonstrated using Raji as the target cells in the presence of rrituximab, an anti‐CD20 antibody clinically used to treat non‐Hodgkin B‐cell lymphoma (Figure 1b, right panel). Notably, co‐culture with activated CD8^+^ T cells also enhanced NK killing efficiency (Figure 1c). This T cell‐boosted NK killing was further confirmed using live‐cell imaging for both natural cytotoxicity (Figure 1d; Movie S1, Supporting Information) and ADCC (Figure 1e; Movie S2, Supporting Information).

Importantly, this T cell‐mediated enhancement in NK cell killing efficiency persisted for at least 7 days (Figure S1a, Supporting Information). Interestingly, the co‐culture period could be shortened to 24 h without compromising the enhancement in NK cell killing (Figure S1b, Supporting Information, compare green to red curve). Further analysis revealed that neither T cells alone, NK cells stimulated by CD3/CD28 beads (Figure S2a, Supporting Information), nor NK cells co‐cultured with unstimulated T cells (Figure S2b, Supporting Information) recapitulated the strong enhancement in NK cell killing observed with activated T cells. Additionally, when 3‐day‐stimulated T cells were simply added to NK and target cells at the start of the killing assay, only a slight increase in NK cell killing efficiency was observed (Figure S2c, Supporting Information), likely due to the previously published bystander effect.^[^ ^22^ ^]^

To further confirm that co‐culturing with T cells enhances NK cell killing capacity, we isolated NK cells from the co‐culture and examined their cytotoxic function. To differentiate between apoptosis and necrosis, we used the K562 cell line stably expressing the apoptosis reporter pCasper, a GFP‐RFP FRET pair linked by a caspase recognition site (DEVD).^[^ ^23^ ^]^ When undergoing apoptosis, K562‐pCasper cells lose the FRET signal and appear green, whereas necrotic cells, with compromised plasma membrane integrity, lose fluorescence entirely. Live‐time imaging revealed that co‐culture with T cells significantly accelerated NK cell killing kinetics (Figure 1f; Movie S3, Supporting Information) without altering the mode of target cell death (apoptosis vsnecrosis, Figure 1g). Moreover, stimulating T cells within PBMCs using CD3/CD28 beads for three days also markedly enhanced NK cell‐mediated killing kinetics in PBMCs (Figure 1h). Taken together, these findings demonstrate that co‐culture with activated T cells substantially boosts NK cell killing efficiency, improving their ability to eliminate tumor cells.

### 2.2 Co‐Culture with Activated T Cells Substantially Enhances NK Cell Motility

To understand the specific changes in NK cells by T cell co‐culture, we first examined NK cell proliferation, viability, and differentiation. Since both CD4^+^ and CD8^+^ T exhibited very similar effects in enhancing NK cell killing kinetics, we focused on CD4^+^ T for further investigation. Our analysis revealed that NK cell proliferation, viability, and subpopulations remained unaffected by T cell co‐culture (Figure S3a—c, Supporting Information). Unexpectedly, the expression of key cytotoxic proteins, such as perforin and granzyme B, was even moderately reduced in NK cells co‐cultured with T cells (Figure S3d,e, Supporting Information). However, this reduction appears to remain within a functional range, as our data show that the boosted NK cells maintained robust killing capacity.

To further investigate alterations in surface receptors and key molecules influenced by T cell co‐culture, we utilized cytometry by time of flight (CyTOF) combined with flow cytometry, enabling the simultaneous assessment of 35 surface and intracellular markers at the protein level. These markers covered: NK cell activating receptors (NKp46, NKp44, NKp30, and NKG2D), inhibitory receptors (NKG2A, KIR2DL2, KIR3DL1, and CD85j/ILT2), cytotoxicity markers (perforin, granzyme B, IFNγ, TNFα), exhaustion markers (T‐bet and TIGIT), adhesion/homing and migration markers (CD54/ICAM‐1, CD11a/LFA‐1, CD69, CD197/CCR7, CD185/CXCR5, CD186/CXCR6, and CX3CR1), cytokine receptors (CD25/IL‐2Rα, CD122/IL‐2Rβ, CD132/IL‐2Rγ, CD126/IL‐6R, and CD127/IL‐7Rα), and general type/subset markers (CD2, CD3, CD4, CD8a, CD16, CD56, CD57, CD117/c‐Kit, and CD161).

**Figure 2:**
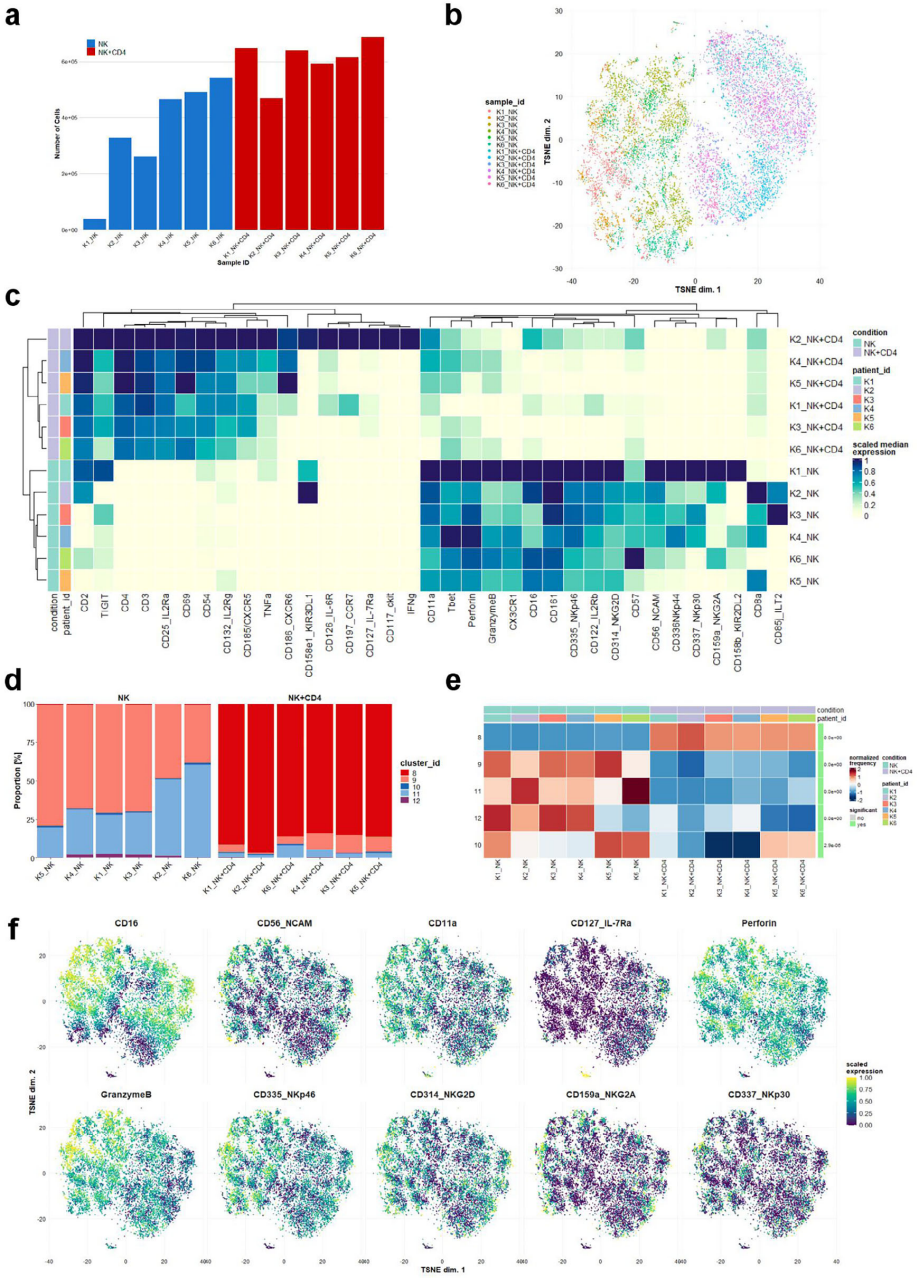
T cell co‐culture induces phenotypic and functional changes in NK cells. Primary NK and CD4^+^ T cells were isolated from six donors. NK cells were either cultured alone (NK) or co‐cultured with autologous bead‐stimulated CD4^+^ T cells (NK+CD4) for 3 days. CyTOF analysis was performed to assess the expression levels of the indicated proteins. a) Number of cells acquired by mass cytometry in each sample. b) t‐SNE showing the distribution of each sample. c) Heatmap showing the expression of all markers in all samples. d) Frequency of the five clusters (8–12) of NK cells in the samples. e) Frequency of clusters and statistical enrichment in the samples according to the conditions (NK vs NK+CD4). f) t‐SNE plots of selected markers showing the expression in NK markers in NK cells alone and following culture with CD4^+^ T cells.

We analyzed primary NK and T cells from six donors, totaling 5.7 × 10^6^ cells, with an average of 480 000 cells per sample (**Figure** **2**a). Using FlowSOM clustering and delta area consensus clustering, 12 clusters were identified and visualized via t‐SNE scatter plot (Figure S4a, Supporting Information). Type/subset marker expression revealed that clusters 1–7 corresponded to CD4^+^ T cells, while clusters 8–12 were NK cells (Figure S4b, Supporting Information). Notably, both NK cells cultured alone and T cell co‐cultured NK cells were distinctively separated across samples (Figure 2b). While NK cells cultured alone expressed their classical markers, NK‐T cell co‐culture displayed markers from both cell types (Figure 2c). To refine the analysis, NK cell‐containing clusters were filtered, and a second t‐SNE dimensionality reduction confirmed distinct clustering based on culture conditions (Figure S4c,d, Supporting Information). Cluster 8, which dominated the dataset, was significantly enriched in co‐culture samples (Figure 2d,e). The analysis revealed a significant upregulation of several NK cell surface molecules with T cell co‐culture, including the activation marker CD69, the IL‐2 receptor α (CD25) and γ (CD132) chains, chemokine receptor CD185/CXCR5, and adhesion molecule CD54/ICAM‐1 (Figure 2c,f; Figure S4e,f, Supporting Information). Notably, a reduction in perforin and granzyme B levels was also observed (Figure 2c,f; Figure S4e,f, Supporting Information).

Intriguingly, we observed an unexpected expression of the T cell marker CD3 and CD4 in T cell co‐cultured NK cells, both in the heatmap (Figure 2c) and the dominating cluster 8 (Figure S4d,e, Supporting Information). To investigate whether cluster 8 represented a heterogeneous population including both NK and T cells, we first examined the expression intensity of CD4 across three clusters: cluster 1 (CD4⁺ T cells), cluster 8 (NK cells after co‐culture), and cluster 9 (NK cells without co‐culture). We found that CD4 expression in cluster 8 was markedly lower than in cluster 1 and was intermediate between clusters 1 and 9 (Figure S5a, Supporting Information), suggesting that cluster 8 is distinct from canonical CD4⁺ T cells. To further test whether cluster 8 represents a mixed population or a distinct NK cell subset with atypical marker expression, we performed a higher‐resolution clustering analysis. Using FlowSOM, we re‐clustered the cells from cluster 8 into eight sub‐clusters and analyzed the expression profiles of key markers (Figure S5b, Supporting Information). All sub‐clusters consistently expressed NK cell–associated markers, including CD16, CD56, CD11a, NKp46, and NKG2D, as well as cytotoxic molecules perforin and granzyme B; some sub‐clusters also showed expression of NKG2A and NKp30 (Figure S5b, Supporting Information). Importantly, all sub‐clusters expressed CD4 to varying degrees, though not all individual cells were CD4⁺. To further distinguish these sub‐clusters from conventional T cells, we analyzed CD4 and CD16 co‐expression. All cluster 8 sub‐clusters were CD16⁺ and exhibited only low levels of CD4 (mean marker intensity < 2), whereas cluster 1 (CD4⁺ T cells) showed strong CD4 expression and no CD16 (mean marker intensity < 1) (Figure S5c, Supporting Information), supporting the conclusion that cluster 8 cells are phenotypically distinct from CD4⁺ T cells.

To determine whether these CD3^+^ NK cells were genuine NK cells, we co‐incubated CFSE‐labeled NK cells with T cells. Flow cytometry indeed detected CD3 signals on the CFSE‐labeled NK cells (Figure S6a,b, Supporting Information), where NK and T cells are clearly distinguishable by CFSE labeling. Thus, the CD3⁺ fraction within the CFSE⁺ population (ranging from ≈6% to 40%) is unlikely to result from T cell contamination. To support these findings, we performed live‐cell imaging with CFSE‐labeled NK cells and CD3‐labeled CD4⁺ T cells. CD3‐positive dots were observed to transfer from T cells to NK cells during contact (Figure S6c, Supporting Information). Together, these data further support the conclusion that the CD3^+^ NK subpopulation represents a distinct subset induced by T cell co‐culture.

**Figure 3:**
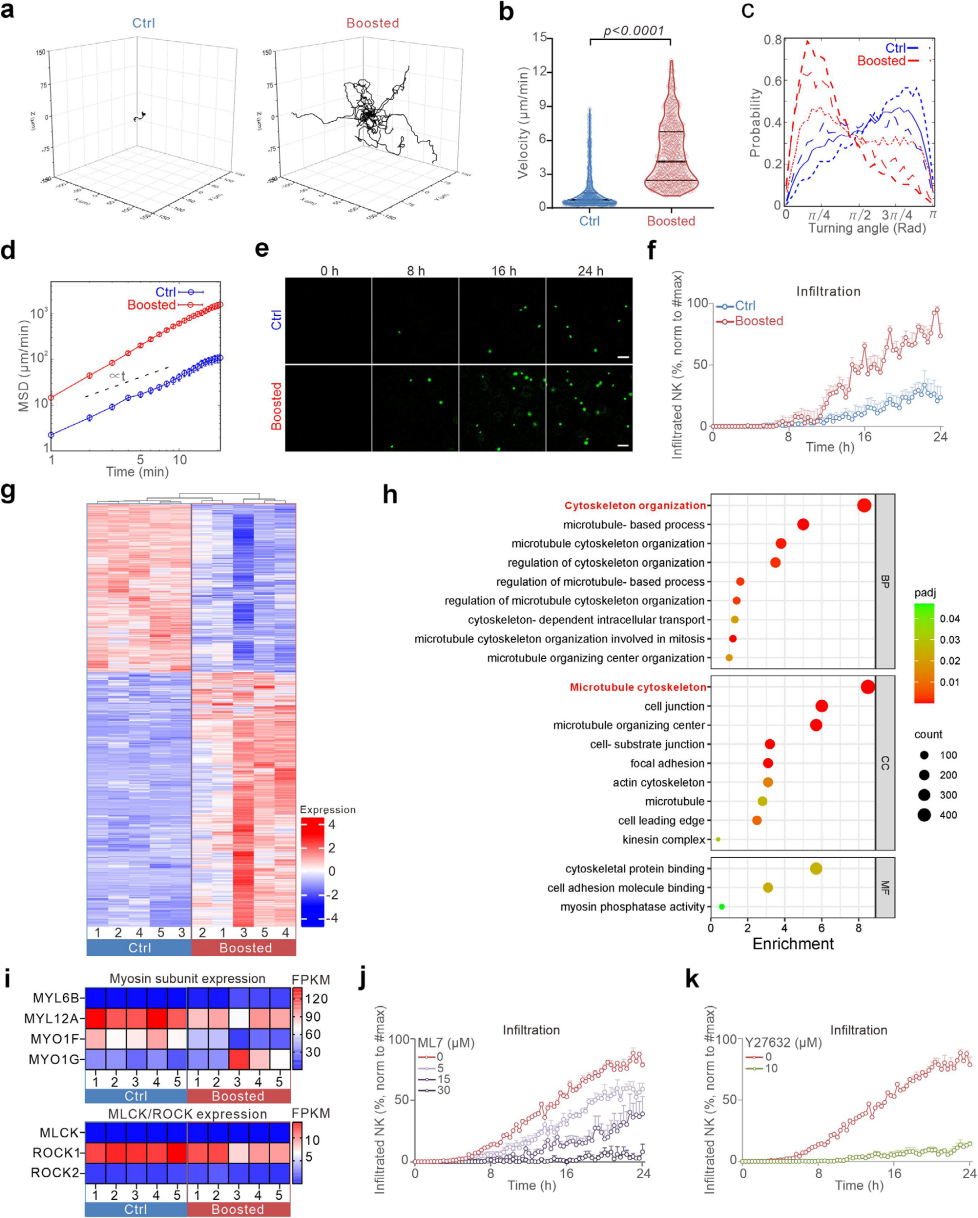
T cell co‐culture enhances NK cell motility. Primary NK cells were either cultured alone for three days (Ctrl) or co‐cultured with autologous bead‐stimulated CD4^+^ T cells on Day 2 post‐isolation for 24 h (Boosted). a–d) NK cell migration in 3D environments. NK cells were loaded with CFSE on Day 1 post‐isolation. For live‐cell imaging, cells were embedded in a collagen matrix (2 mg mL^−1^) and NK cell movements were visualized via light‐sheet microscopy (20× objective) every 30 s for 60 min at 37 °C. NK cells were tracked using Imaris. Trajectories (n = 30 cells for each condition) are shown in a. Quantification of migration velocity (b), turning angles (c), and MSD (d) is shown (Ctrl: n = 574 cells; Boosted: n = 597 cells). All results are from 4 donors. Mann–Whitney U‐test was conducted for b. e,f) NK cell infiltration into collagen matrices. Cells were put from top of a collagen matrix (2 mg mL^−1^), and images were taken at the bottom layer every 20 min for 24 h at 37 °C using high‐content imaging (ImageXpress, 20× objective). Time‐lapse images of a representative donor are shown (e), with quantification in f (mean ± SEM, normalized to the maximum recorded infiltrated cell number, n = 4 donors). g–i) Transcriptomic changes in NK cells following T cell co‐culture. NK cells were isolated with CD56 magnetic beads from NK‐T co‐culture (Boosted) or NK cultured alone (Ctrl), and RNA‐seq was performed (n = 5 donors). Differentially expressed genes (g), Gene Ontology (GO) enrichment for cytoskeleton/migration‐related pathways (h), and changes in expression of myosin subunits, MLCK, and ROCK (i) are shown. j,k) Myosin pathways regulate NK cell infiltration. NK cells were put from the top of a collagen matrix (2 mg mL^−1^) with ML‐7 (j) or Y‐27632 (k) present in the media. Images were acquired at the bottom layer every 20 min for 24 h at 37 °C using high‐content imaging (ImageXpress, 20× objective). Quantification of infiltrated NK cells is shown as mean ± SEM (normalized to the maximum recorded infiltrated cell number, n = 4 donors).

Given that efficient migration is crucial for NK cells to locate and eliminate target cells, we next examined their migration. To simulate a physiologically relevant environment, we embedded NK cells in a 3D collagen matrix and tracked their movement using light‐sheet microscopy. We observed that T cell co‐cultured NK cells exhibited substantially increased motility compared to NK cells alone (**Figure** **3**a; Movie S4, Supporting Information). Both migration velocity and the fraction of highly mobile NK cells (velocity > 2 µm min^−1^) were markedly enhanced by T cell co‐culture (Figure 3b). Furthermore, NK cell migration persistence, defined by the directionality of movement, was significantly increased, as evidenced by reduced turning angles (Figure 3c) and elevated mean square displacements (MSD) (Figure 3d). In addition, we assessed the infiltration capability of NK cells into 3D matrices by seeding NK cells on top of a collagen matrix. Live cell imaging revealed that NK cells co‐cultured with T cells reached the bottom layer earlier and in greater numbers compared to controls (Figure 3e,f), indicating that T cell co‐culture enhances NK cell infiltration into 3D environments.

To explore the molecular mechanism responsible for T cell‐enhanced NK migration and infiltration, we performed RNA sequencing (Figure 3g). Gene Ontology (GO) enrichment analysis of migration‐related pathways revealed that genes involved in cytoskeleton organization and microtubule cytoskeleton were greatly impacted by T cell co‐culture (Figure 3h). Notably, several myosin subunits, including MYL12A, MYO1F, and MYO1G, exhibited substantial expression changes (Figure 3i). Myosin activity is spatially regulated by myosin light chain kinase (MLCK) and Rho‐associated protein kinase (ROCK).^[^ ^24^ ^]^ Both MLCK (also known as MYLK1) and ROCK1 are expressed in NK cells, as confirmed by RNA‐Seq analysis (Figure 3i). To investigate their roles in T cell‐boosted NK motility, we pharmacologically inhibited MLCK (using ML7) or ROCK (using Y‐27632) and assessed NK infiltration into the 3D matrix. Inhibition of either MLCK or ROCK significantly reduced the number of T cell co‐cultured NK cells that successfully penetrated the collagen matrix and reached the well bottom (Figure 3j,k). Collectively, these findings suggest that T cell‐enhanced NK functionality is primarily mediated by increased myosin‐dependent migration and infiltration, rather than increased cytotoxic granule content.

### 2.3 T Cell Co‐Culture Restores Cryopreservation‐Impaired NK Killing Efficiency

**Figure 4:**
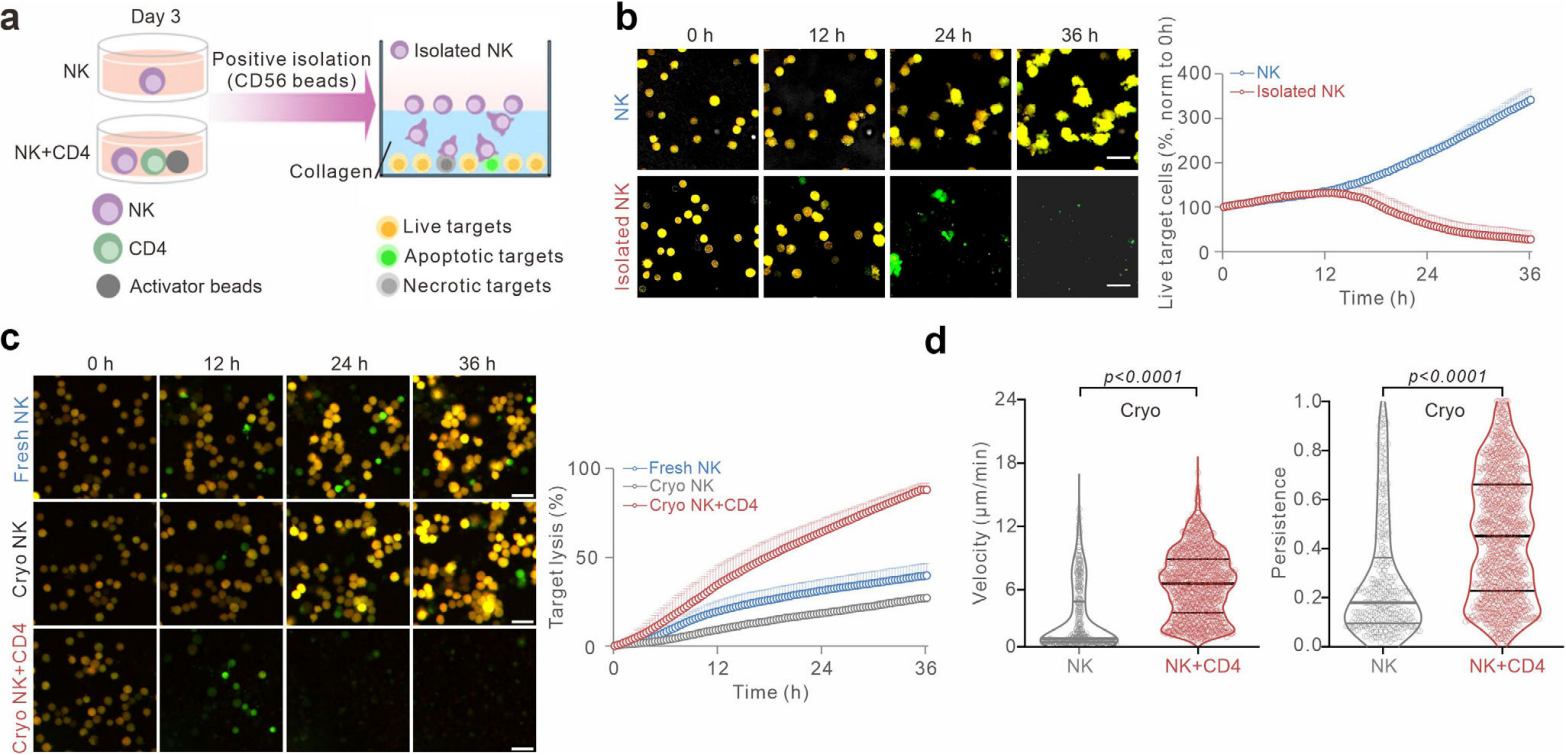
T cell co‐culture rescues cryopreservation‐impaired NK cell functions. a,b) NK cell killing kinetics in 3D. (a) NK cells were isolated with CD56 magnetic beads from NK‐T co‐culture (Isolated NK) or NK cultured alone (NK). (b) Killing efficiency against K562‐pCasper target cells (E:T = 2.5:1) was determined via a 3D real‐time killing assay using high‐content imaging (ImageXpress, 20× objective). Scale bars: 40 µm. Quantification of live target cells is shown as mean ± SEM (normalized to Time 0, n = 4 donors). c,d) Restoring cryopreserved NK cell functionality via T cell co‐culture. After thawing, cryopreserved NK cells were either co‐cultured alone (Cryo NK) or with autologous bead‐activated CD4⁺ T cells (Cryo NK+CD4). Freshly isolated primary NK cells (Fresh NK) were used as controls. Killing efficiency in 3D (c) was assessed via a 3D real‐time killing assay and quantification is shown as mean ± SEM (n = 4 donors). For 3D migration analysis (d), NK cells were labeled with CFSE before co‐culturing with T cells. Cells were embedded in collagen (2 mg mL^−1^) and imaged using light‐sheet microscopy (20× objective). Quantification is shown as violin plots with median and inter‐quartile range (NK: n = 332 cells, NK+CD4: n = 1058 cells, from 4 donors). Mann–Whitney U‐test was conducted.

In vivo, one major challenge that NK cells encounter is locating and eliminating tumor cells in complex 3D environments. To assess whether T cell‐boosted NK cells also exhibited enhanced killing efficiency in 3D, we used a 3D killing assay established in our lab,^[^ ^25^ ^]^ in which K562‐pCasper cells were embedded in a 3D collagen matrix, and after the collagen solidified, NK cells were added from above, allowing them to infiltrate, search, and attack target cells in a physiologically relevant setting (**Figure** **4**a). To rule out possible influence from T cells, we isolated NK cells from the T‐NK co‐culture. Live‐cell imaging over 36 h revealed that T cell co‐cultured NK cells initiated killing earlier than NK cells cultured alone, and by 24 h NK cells from T‐NK co‐culture efficiently eliminated nearly all tumor cells through apoptosis and necrosis, whereas unboosted NK cells failed to control K562 proliferation (Figure 4b; Movie S5, Supporting Information). These findings indicate that in a 3D environment, T cell‐boosted NK cells possess superior tumor‐clearing ability.

NK cells are widely used in immunotherapy, and significant efforts are underway to optimize treatments using CAR‐NK cells, NK‐92 cells, iPSC NK cells, CIML NK cells, adoptive NK cells, and UCB HPC‐derived NK cells.^[^ ^26^ ^]^ To ensure the efficient application of these therapeutic strategies, cryopreservation of NK cells is often required. However, previous studies ^[^ ^18^ ^]^ have shown that cryopreservation significantly impairs NK cell migration and cytotoxicity in 3D environments. We tested whether T cell co‐culture could restore this impaired function. Particularly, after thawing cryopreserved primary NK cells, they were co‐cultured with activated T cells for 24 h prior to experiments. Live‐cell imaging from the 3D killing assay shows that while control NK cells were unable to control tumor cell proliferation, T cell‐boosted NK cells eradicated nearly all tumor cells within 36 h (Figure 4c), and they exhibited substantially enhanced migration velocity and persistence (Figure 4d). Collectively, these findings highlight T cell co‐culture as a powerful strategy to rescue cryopreservation‐impaired NK cell function.

### 2.4 Physical Contact between NK and T Cells is Essential for Boosting NK Cell Function via IL‐2

**Figure 5:**
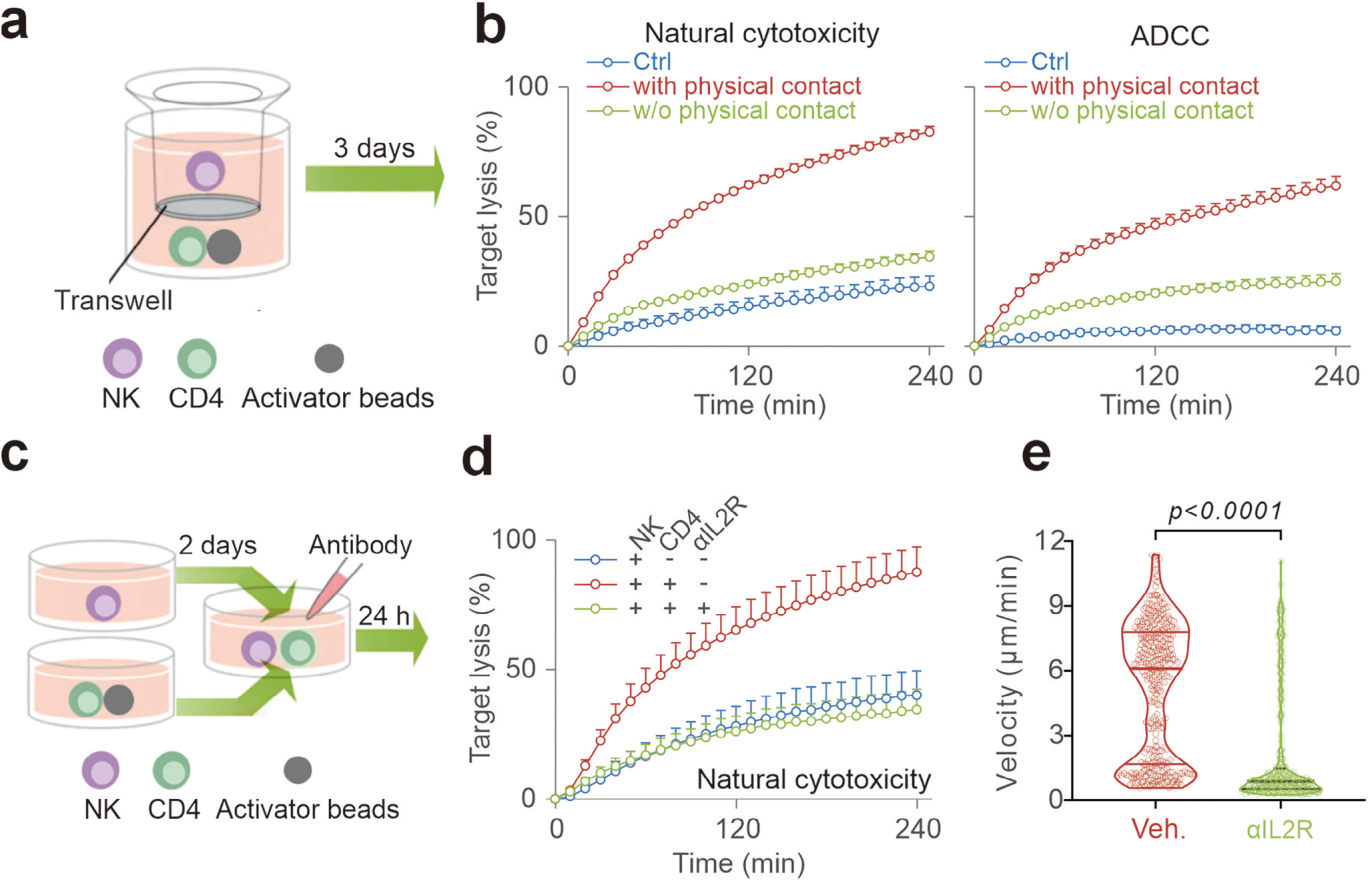
Direct contact and IL‐2 signaling are essential for T cell‐boosted NK cell function. a) Schematic of the transwell experiment. NK cells were cultured for three days in a 0.4 µm transwell insert, with CD4⁺ T cells and beads seeded in the outer well. b) T cell‐mediated enhancement of NK cytotoxicity requires direct contact. NK cell killing kinetics were measured using a plate reader‐based 2D real‐time killing assay. K562 (for natural cytotoxicity) and Raji (for ADCC) cells were used as target cells (E:T = 2.5:1). Ctrl: NK cells alone; with physical contact: NK‐T co‐culture; w/o physical contact: NK cells from transwell. Data are shown as mean ± SEM (n = 4 donors). c–e) IL2 signaling blockade abolishes T cell‐boosted NK cell functionality. IL‐2 signaling was inhibited by basiliximab treatment (αIL2R, 5 µg mL^−1^) as depicted in c. NK cell killing kinetics (d) was determined using a plate reader‐based 2D real‐time killing assay with K562 as target cells (E:T = 2.5:1). Quantification is shown as mean ± SEM (n = 4 donors). NK cell movement in 3D (e) was visualized via light‐sheet microscopy (20× objective). Quantification is shown as violin plots with median and inter‐quartile range (Veh: n = 197 cells, αIL2R: n = 219 cells, from 2 donors). Mann–Whitney U‐test was conducted.

A key question arising from our findings is how co‐culture with activated T cells enhances NK cell killing efficiency. Since T cell‐derived cytokines are known to activate various immune cells, including NK cells, we hypothesized that T cell‐secreted cytokines play a critical role in boosting NK cell function. To test this hypothesis, we employed a transwell system to physically separate T cells and NK cells while still allowing NK cells access to T cell‐released cytokines (**Figure** **5**a). Surprisingly, regardless of whether NK cells were in the insert or the outer well, the presence of activated T cells only marginally increased NK cell killing efficiency, far below the enhancement observed when NK cells had physical contact with T cells, and this effect was consistent for both natural cytotoxicity and ADCC (Figure 5b). To ensure that NK cells had full access to secreted cytokines, we manually mixed the supernatant between the insert and outer well, and this did not improve NK cell killing efficiency (Figure S7, Supporting Information). These results indicate that physical contact between NK and T cells is indispensable for enhancing NK killing function.

Given that direct contact was required, we initially postulated that IL‐2 was not the key mediator. To test this, we blocked IL‐2 signaling using basiliximab, a neutralizing antibody against IL‐2 receptor α chain CD25, during the 24 h‐co‐culture period (Figure 5c). Unexpectedly, in the presence of basiliximab (αIL2R), the T cell‐mediated enhancement of NK killing efficiency against K562 cells was completely abolished, reducing NK cell killing to baseline levels observed in NK cells cultured alone (Figure 5d). Moreover, NK cell mobility in 3D environments was also significantly impaired (Figure 5e). These results suggest that T cell‐derived IL‐2 is indeed required for boosting NK cell killing function.

If IL‐2 is essential, it is reasonable to expect that adding recombinant IL2 should replicate the enhancement seen with T cell co‐culture. To test this, we first quantified IL‐2 levels in the supernatant using a flow cytometry‐based multiplex cytokine assay. We found that IL‐2 concentrations in the supernatant from a 3‐day T‐NK co‐culture were comparable to those from 3 day‐stimulated CD4^+^ T cells (Figure S8a, Supporting Information). In contrast, IL‐2 levels in NK alone cultures were minimal, similar to 1 day co‐culture conditions, and barely above the detection limit (Figure S8a, Supporting Information). To determine the equivalent concentrations for IL‐2, we cultured NK cells with 0.5, 5, and 50 ng mL^−1^ of IL‐2 for three days and measured IL‐2 levels in the supernatant. We found that 50 ng mL^−1^ IL‐2 closely matched IL‐2 concentrations in 3‐day co‐culture, while 0.5 ng mL^−1^ IL‐2 was comparable to 1‐day co‐culture levels. NK cells cultured in the presence of 0.5 ng mL^−1^ IL‐2 for 1 day did not exhibit enhanced cytotoxicity (Figure S8b, Supporting Information), nor did they after 3 days of IL‐2 treatment (Figure S8c, Supporting Information). Even at 50 ng mL^−1^ IL‐2, NK cell killing remained unchanged after 1 day‐stimulation (Figure S8c, Supporting Information). Culturing NK cells with supernatant from 3‐day stimulated T cells also failed to elevate NK cell killing kinetics (Figure S8d, Supporting Information). These data suggest that NK cell killing is not enhanced by IL‐2 freely diffusing in the supernatant, but rather by IL‐2 locally released at the NK‐T contact site, where it likely reaches a critical threshold necessary for boosting NK cell function.^[^ ^27^, ^28^ ^]^

### 2.5 Synthetic IL‐2‐Presenting Cells Effectively Enhance NK Cell Killing Efficiency

**Figure 6:**
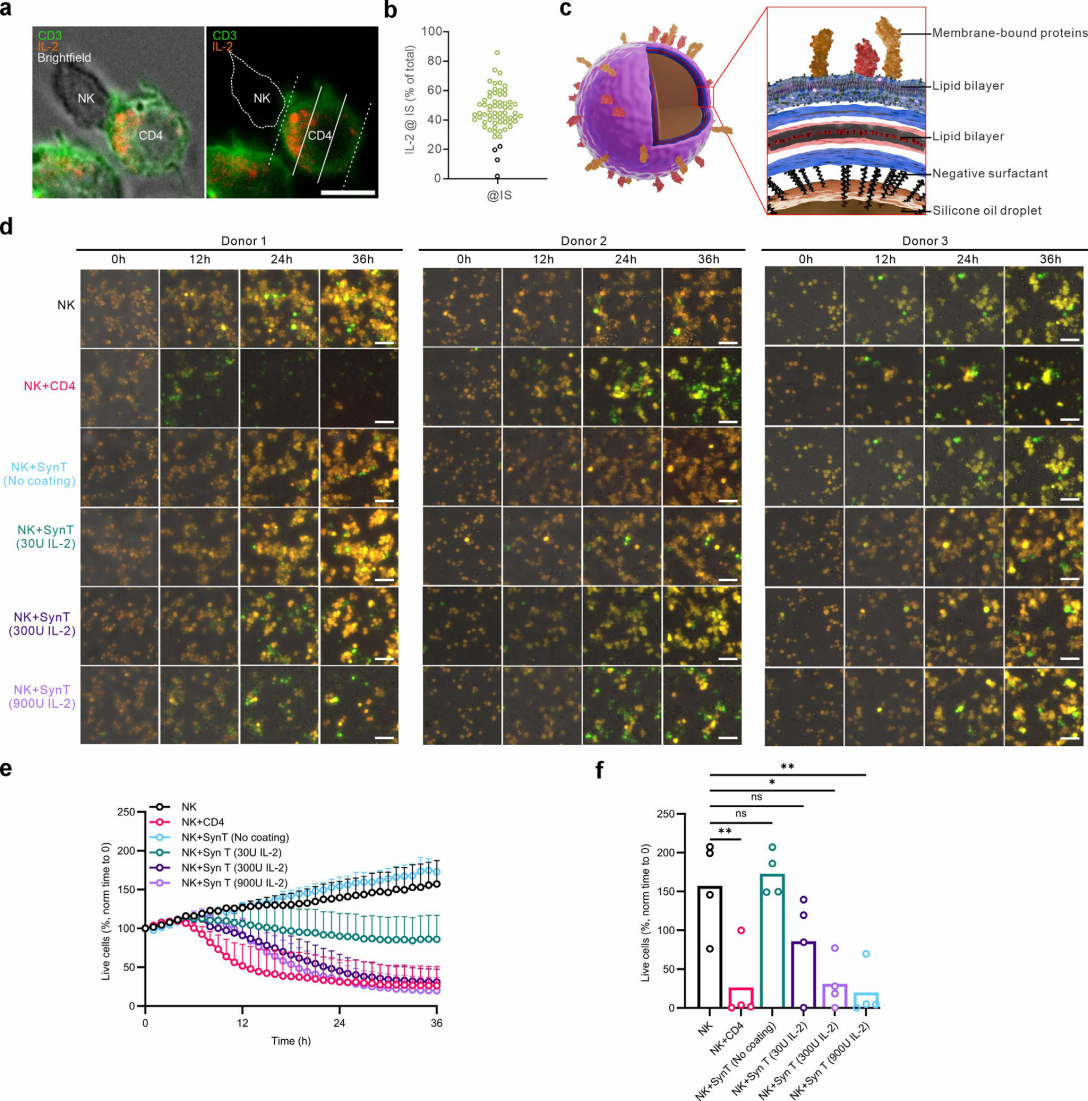
Synthetic IL‐2 presenting T cells restore cryopreservation‐impaired NK killing efficiency. a,b) Endogenous IL‐2 accumulates at the NK‐T cell contact sites. NK and CD4⁺ T cells were seeded on fibronectin‐coated coverslips and immunostained to visualize endogenous IL‐2 localization. CD3 was used as a T cell marker. Imaging was performed using a Cell Observer with a 60× objective. The contact site vicinity was defined as the region within one‐third of the cell diameter closest to the contact site (≈26% of total cellular volume). Scale bars: 5 µm. Images from a representative conjugation are shown. Quantification of IL‐2 fraction near the contact site is shown in b, with green circles marking the values > 26%. c–f) Synthetic IL‐2 presenting T cells rescue NK cell killing efficiency. Schematic representation of IL‐2 presenting synthetic cells is depicted in c. NK cells were co‐cultured for 24 h with synthetic cells (SynT) with surface‐bound IL‐2 (30, 300, 900U) or with autologous bead‐stimulated CD4^+^ T cells. NK cells killing kinetics was assessed using the 3D real‐time assay (E:T = 2:1) and visualized via high‐content imaging (ImageXpress, 20× objective). Time‐lapse image series of three donors are shown (d, scale bars: 40 µm) with kinetics across all time points (e, mean ± SEM) and quantification at the end point (36 h) (f, single values with means). Results are from 4 donors. Statistical analysis was conducted via a two‐way ANOVA with multiple comparisons (e) or one‐way ANOVA with Tukey's multiple comparisons (f).

To further investigate the spatial localization of IL‐2, we examined IL‐2 distribution in T cells in contact with NK cells. Immunostaining analysis revealed that endogenous IL‐2 predominantly accumulated in the vicinity of the NK‐T contact site (**Figure** **6**a), with significantly higher density (Figure 6b, green circles) than expected by random distribution (Figure 6b, black circles). This finding led us to hypothesize that high local IL‐2 concentration at the contact site plays a critical role in boosting NK cell function. To test this, we used synthetic cells, engineered particles with a silicon core coated by a lipid bilayer, where purified recombinant IL‐2 was covalently linked to the lipid surface (Figure 6c). We generated synthetic cells with three IL‐2 concentrations, corresponding to a total amount of 30, 300, and 900 U being used to decorate 3 × 10^6^ particles. Primary NK cells were co‐cultured with these IL‐2‐presenting synthetic cells for one day, with bead‐activated autologous T cells as a positive control. Using the 3D real‐time killing assay, we analyzed NK cell cytotoxicity after 24 h‐co‐culture with synthetic cells. Live cell imaging revealed that NK cell killing efficiency in 3D was significantly enhanced at higher concentrations (300 and 900 U), reaching levels comparable to those observed with T cell co‐culture, whereas at the lowest concentration (30 U), only an insignificant increase was observed, while non‐coating control synthetic cells did not alter NK cell killing efficiency (Figure 6d–f; Movie S6, Supporting Information). These findings highlight synthetic IL‐2‐presenting cells as a promising approach to mimic T cell‐mediated NK activation, offering a potential tool for enhancing NK cell‐based immunotherapies.

IL‐15 is widely used to support NK cell expansion and has also been engineered into CAR‐NK cells to improve their viability and persistence in vivo.^[^ ^29^, ^30^ ^]^ To assess whether soluble IL‐15 can replicate the boosting effects provided by our co‐culture strategy, we stimulated NK cells with IL‐15 at three concentrations (10, 30, and 100 U mL^−1^). After 24 h, all three doses enhanced NK cell killing efficiency, but the effect remained significantly lower than that achieved through co‐culture with CD4^+^ T cells under both 2D (Figure S9a, Supporting Information) and 3D conditions (Figure S9b—e, Supporting Information). Interestingly, combining IL‐15 with IL‐2 slightly reduced NK cell cytotoxicity compared to IL‐15 alone under both 2D (Figure S9a, Supporting Information) and 3D settings (Figure S9b—e, Supporting Information), possibly due to competition for the shared γ‐chain in their receptor complexes. These findings indicate that our 1‐day co‐culture strategy provides a more potent approach to boosting NK cell cytotoxicity than stimulation with soluble IL‐15.

**Figure 7:**
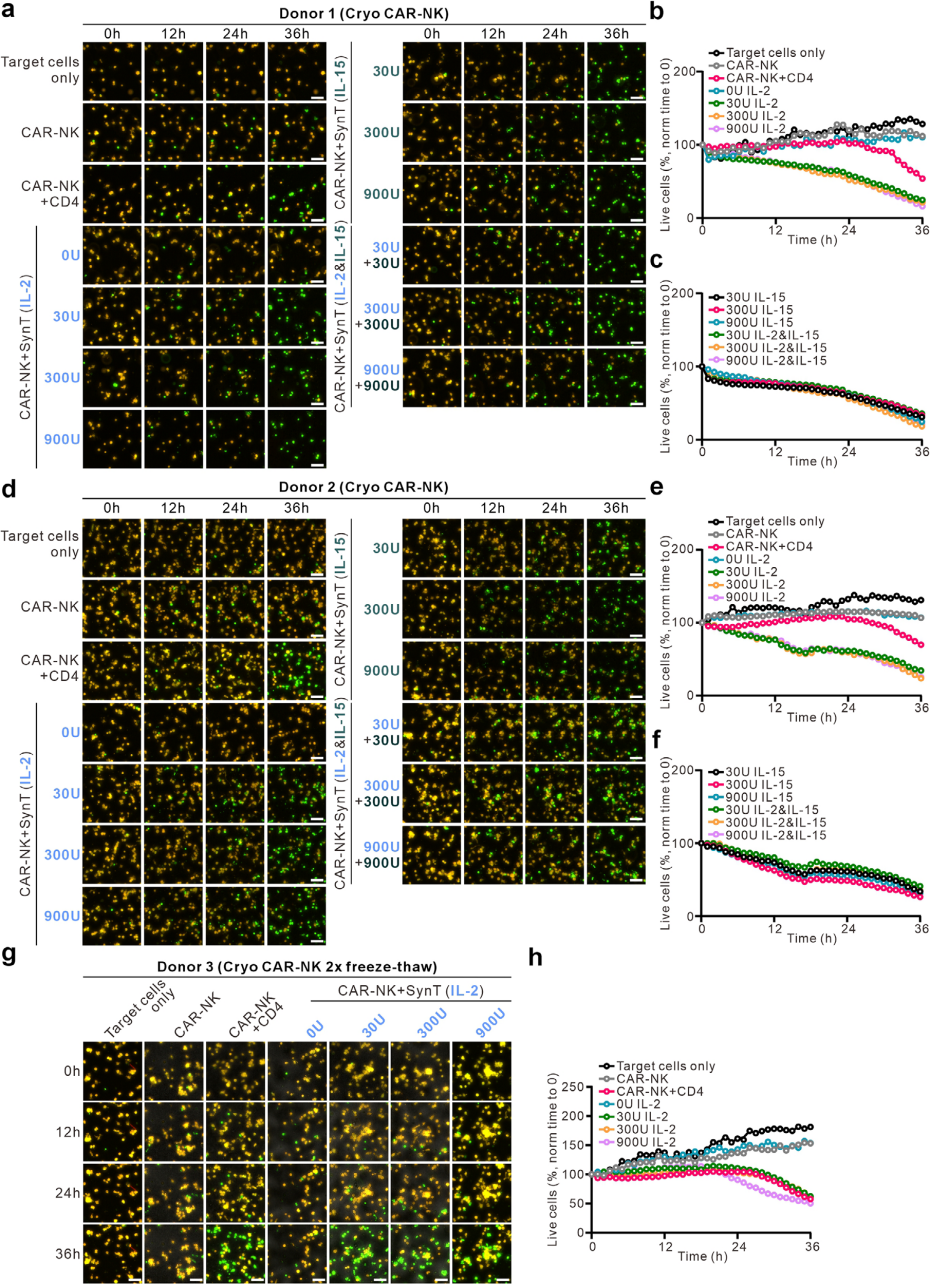
Both IL‐2‐ and IL‐15‐presenting synthetic cells can restore cryopreserved CAR‐NK cell cytotoxicity in 3D. CAR‐NK cells targeting CD19 were either directly thawed (a–f) or underwent expansion after thawing followed by a second freeze–thaw cycle (g–h). NK cells were co‐cultured for 24 h with either synthetic cells (SynT) or allogeneic CD4⁺ T cells activated with CD3/CD28 beads. SynT were surface‐coated with IL‐2, IL‐15, or a mixture of both (IL‐2+IL‐15 condition used a 1:1 mix of IL‐2– and IL‐15–coated SynT). TMD8‐pCasper tumor cells were used as targets, and NK cell cytotoxicity was evaluated using a 3D real‐time killing assay (E:T ratio = 2.5:1) using high‐content imaging (ImageXpress, 20× objective). Representative time‐lapse images are shown for two donors for one freeze‐thaw cycle (a, d) and one donor for a double freeze‐thaw scenario (g) with scale bars of 40 µm. Quantitative killing kinetics across all time points are shown in panels b–c (for a), e–f (for d), and h (for g).

To demonstrate the clinical relevance and translational potential of our approach, we tested cryopreserved CD19‐targeting CAR‐NK cells obtained from two donors. Expression of CD19‐targeting CAR was verified using flow cytometry (Figure S10, Supporting Information). Using 3D killing assays, we found that a 24‐h co‐culture with IL‐2‐presenting synthetic cells substantially enhanced the cytotoxic activity of cryopreserved CAR‐NK cells against TMD8 target cells (CD19^+^ human B lymphoma cells) from both donors — reaching levels comparable to, or even exceeding, those achieved through CD4^+^ T cell co‐culture (**Figure** **7**a–f). In addition, we also assessed the potential of IL‐15‐presenting synthetic cells on boosting cryopreserved CAR‐NK functions. Using the 3D killing assay, we observed that IL‐15–presenting synthetic cells enhanced CAR‐NK cytotoxicity to similar levels as that of IL‐2‐presenting cells (Figure 7a–f). A combination of IL‐2 and IL‐15 synthetic cells did not further enhance CAR‐NK function (Figure 7a–f). These data suggest both IL‐2‐ and IL‐15‐presenting synthetic cells are effective tools for restoring the cytotoxic function of cryopreserved CAR‐NK cells.

To simulate a clinically relevant off‐the‐shelf scenario—where CAR‐NK cells are thawed, expanded, and re‐cryopreserved—we applied a double freeze‐thaw protocol. Following this, CAR‐NK cells exhibited minimal cytotoxicity (Figure 7g,h). However, after 24‐h co‐culture with IL‐2–presenting synthetic cells, even at the lowest cytokine dose (30 U mL^−1^), cytotoxicity was fully restored, with nearly complete elimination of TMD8 target cells (Figure 7g,h). This represented a more than 10‐fold increase in killing capacity, comparable to that achieved with activated CD4⁺ T cells (Figure 7g,h). Importantly, synthetic cells without IL‐2 coating had no boosting effect across all donors (Figure 7a–h). Together, these results demonstrate the robust capacity of IL‐2–presenting synthetic cells to restore and enhance the cytotoxic function of cryopreserved CAR‐NK cells, underscoring their translational promise for future off‐the‐shelf NK cell‐based immunotherapies.

## 3 Discussion

We demonstrate that co‐culturing NK cells with activated T cells for just 24 h significantly enhances NK cell motility and killing efficiency, particularly in 3D environments. Notably, this approach effectively restores the impaired 3D cytotoxic functionality of cryopreserved NK cells. Our findings reveal that T cells deliver IL‐2 at T‐NK contact sites, rather than by freely diffusing IL‐2 in the extracellular space. This is further supported by the superior boosting effect from synthetic cells engineered with surface‐bound IL‐2. Moreover, these synthetic IL‐2‐presenting cells offer an innovative and universal approach to enhance the functionality of NK cells, including cryopreserved CAR‐NK cells. This strategy offers a powerful tool to restore NK potency after cryopreservation, which is particularly crucial for improving the efficacy of off‐the‐shelf NK cell‐based therapies.

Off‐the‐shelf NK cell‐based immunotherapies typically require cryopreservation of the final drug product in GMP‐grade bags.^[^ ^31^, ^32^ ^]^ However, post‐thaw functional impairment of NK cells remains a known limitation.^[^ ^33^, ^34^ ^]^ Our study demonstrates that brief co‐culture with IL‐2‐presenting synthetic cells can restore, and even enhance, NK cell cytotoxicity post‐thaw, offering a promising strategy to overcome this limitation. To enable clinical translation, GMP compatibility is required. We propose a possible GMP‐compliant workflow to implement this approach: synthetic cells can be engineered with magnetic cores to allow for automated removal under sterile conditions. After thawing, NK cells stored in GMP‐compliant cryobags will be transferred into a closed‐system GMP bioreactor (e.g., CliniMACS Prodigy), where they will first undergo a washing step to remove the cryopreservation medium and will be resuspended in GMP‐grade culture medium supplemented with 5% human AB serum, then co‐cultured with GMP‐grade synthetic cells for ≈24 h. Following this co‐culture, the synthetic cells will be efficiently removed via magnetic depletion (e.g., CliniMACS separation tubing set), ensuring the final product consists solely of functionally enhanced NK cells. The NK cells will then be harvested into a sterile final formulation bag, suspended in 0.9% NaCl containing 0.5% human serum albumin, ready for clinical use. The restored NK cells will then be collected into a sterile infusion bag, suspended in 0.9% NaCl supplemented with 0.5% human serum albumin, and made ready for clinical administration. This process ensures full GMP compliance, avoids open handling, and supports in‐process quality control steps such as sterility, depletion efficiency, and phenotype/function assessment. We acknowledge that this re‐culture step introduces additional complexity compared to direct infusion of cryopreserved drug products, including the need for renewed GMP handling and release testing. Nevertheless, in clinical scenarios where robust NK cell cytotoxicity is essential, such as in patients with aggressive or refractory malignancies, this brief reactivation step may offer a valuable trade‐off. In such high‐risk settings, maximizing cell functionality could critically impact therapeutic outcomes and justify the additional manufacturing effort.

Beyond IL‐2, other cytokines such as IL‐15 have been widely explored for enhancing NK cell functionality.^[^ ^35^, ^36^ ^]^ While particularly CD4^+^ but also CD8^+^ T cells secrete large amounts of IL‐2 upon activation, IL‐15 is typically not produced by T cells.^[^ ^37^, ^38^ ^]^ IL‐2 stimulation is well‐known to enhance NK cell cytotoxic protein expression, degranulation, and target cell killing.^[^ ^23^, ^24^ ^]^ In contrast, IL‐15 drives NK cells toward a memory‐like phenotype, which extends their life span and allows for sustained cytotoxic activity over time.^[^ ^39^ ^]^ IL‐15 has been engineered into CAR‐NK cells to improve their viability and persistence in vivo.^[^ ^29^, ^30^ ^]^ In our study, a 24‐h stimulation with soluble IL‐15 enhanced NK cell killing efficiency, but did not reach the levels achieved through co‐culture with CD4^+^ T cells or IL‐2‐presenting synthetic cells. Remarkably, IL‐15‐presenting synthetic cells significantly boosted the cytotoxicity of cryopreserved CAR‐NK cells, reaching levels comparable to those achieved with IL‐2‐presenting counterparts. These findings highlight the potential of cytokine‐coated synthetic cells as powerful tools to enhance cytokine‐mediated immune cell functions.

A previous study in a mouse model has demonstrated that depletion of CD4^+^ T cells impairs NK cytotoxicity, an effect that could be rescued by IL‐2 administration.^[^ ^40^ ^]^ Consistent with this, multiple studies have reported that the presence of CD4^+^ T cells greatly enhances NK killing capacity.^[^ ^41^, ^42^, ^43^ ^]^ However, our findings reveal that the mere presence of CD4^+^ T cells is not sufficient to boost NK cell function — physical contact between NK and T cells is essential. Furthermore, we show that the cytotoxic CD8^+^ T cells can also play a “helper” role in enhancing NK‐mediated killing.

IL‐2 plays a pivotal role in T cell‐boosted NK cell killing functionality, and blocking IL‐2 receptor signaling completely abolishes this. However, exposure to soluble IL‐2 at a concentration of 50 ng mL^−1^, levels only reached during cytokine storms in vivo,^[^ ^44^ ^]^ for 24 h fails to enhance NK cytotoxicity. These results indicate that while IL‐2 is necessary, it is not sufficient for rapid NK activation. Moreover, high‐dose IL‐2 administration is associated with severe side effects, including the vascular leak syndrome, as observed in cancer patients treated by IL‐2‐based immunotherapy.^[^ ^45^, ^46^ ^]^ Physical contact between T and NK cells provides a localized environment where NK cells can receive high concentrations of IL‐2 without affecting the neighboring non‐target cells. Additionally, LFA‐1/ICAM‐1 interactions between NK and target cells are required to seal the IS, preventing cytotoxic protein leakage and potential damage to neighboring non‐target cells.^[^ ^4^ ^]^ This suggests that LFA‐1 may also contribute to the IL‐2‐mediated boosting effect by maintaining a high local IL‐2 concentration in the cleft at the NK‐T cell interface.

Our study highlights the significant advantages of using synthetic IL‐2‐presenting cells to enhance NK function. Unlike primary T cells, which require activation and are subject to donor variability, synthetic cells provide a standardized, controlled, and reproducible platform for NK cell stimulation. By presenting IL‐2 on their surface, these synthetic cells mimic the localized IL‐2 delivery seen in NK‐T cell interactions, ensuring efficient NK activation while minimizing off‐target effects. Furthermore, synthetic cells eliminate the need for viable donor‐derived T cells, overcoming logistical and scalability challenges in clinical applications. This approach is particularly beneficial for off‐the‐shelf NK cell therapies, where rapid, controlled activation is crucial. Importantly, our in vitro 3D killing assay confirms that synthetic cell‐boosted NK cells can effectively eradicate tumor cells, even in cases where NK cells alone fail to control tumor cell proliferation. This innovative strategy not only enhances the therapeutic potential of NK cells but also lays the foundation for developing novel immunotherapies that harness the power of synthetic cell‐based immune modulation.

In summary, using primary human cells, we identify both CD4^+^ and CD8^+^ T cells as potent enhancers of NK killing functionality, primarily by increasing NK motility. Physical contact between NK and T cells is crucial for this enhancement, with IL‐2 functioning in tandem to mediate the boosting effect. This strategy can also efficiently restore impaired cytotoxicity of cryopreserved NK cells, underscoring the importance of NK‐T cell interactions in optimizing NK cell‐based immunotherapy. Furthermore, we establish synthetic IL‐2‐presenting cells as a powerful tool for overcoming cryopreservation‐induced functional impairments and improving NK cell potency, providing a donor‐independent, scalable, and clinically viable solution to advance NK cell‐based immunotherapy.

## 4 Experimental Section

### Antibodies and Reagents

All chemicals, if not particularly indicated, were from Sigma–Aldrich (highest grade). The following reagents were purchased from ThermoFisher Scientific: human IL‐2 recombinant protein, Calcein‐AM, CellTrace CFSE Cell Proliferation Kit, PBS, and propidium iodide. The following antibodies were purchased from Biolegend: Alexa Flour 488 anti‐human CD16 (Clone:3G8), PerCP anti‐human CD16 (Clone:B73), PerCP anti‐human CD3 (Clone: HIT3a), APC/Cy7 anti‐human CD3 (Clone:SK7), Brilliant Violet 421 anti‐human CD3 (Clone:UCHT1), Brilliant Violet 421 anti‐human CD107a (LAMP1) (Clone:H4A3), PE anti‐human Granzyme B (Clone:QA16A02), Alexa Flour 647 anti‐human Perforin (Clone:dG9), PE anti‐human CD178 (Fas‐L) (Clone:NOK‐1), and Alexa Flour 647 anti‐human IL‐2 (Clone:MQ1‐17H12). APC anti‐human CD56 was from BD Biosciences. Following inhibitory antibodies were used: Efalizumab (anti‐Integrin alpha‐L (ITGAL) antibody, from Antibodies‐online GmbH), basiliximab (IL2RA recombinant monoclonal antibody, from Biozol GmbH). Bovine Type I Collagen Solution (10 mg mL^−1^) was from Advanced BioMatrix. Human IL‐15 was purchased from Miltenyi Biotech.

### Cell Culture

Peripheral blood mononuclear cells (PBMCs) were obtained from healthy donors as previously described.^[^ ^21^ ^]^ Primary human NK, CD4^+^ T and CD8^+^ T cells were negatively isolated from PBMCs according to the manufacturer's instructions using NK Cell Isolation Kit human (Miltenyi Biotec), CD4^+^ T Cell Isolation Kit human (Miltenyi Biotec), CD8^+^ T Cell Isolation Kit human (Miltenyi Biotec), respectively. NK and T cells were cultured in AIM V medium (ThermoFisher Scientific) with 10% FCS and 1% Penicillin‐Streptomycin (ThermoFisher Scientific) at a density of 2 × 10^6^ and 3 × 10^6^ cells mL^−1^. T cells were stimulated with Dynabeads Human T‐Activator CD3/CD28 (ThermoFisher Scientific) for 2 days at a bead‐to‐cell ratio of 0.8:1.

K562, Raji, K562‐pCasper, and TMD8‐pCasper cells were cultured in RPMI‐1640 medium (ThermoFisher Scientific) containing 10% FCS (ThermoFisher Scientific) and 1% Penicillin‐Streptomycin (ThermoFisher Scientific). K562‐pCasper cells were additionally supplemented with 1.25 mg mL^−1^ G418 (ThermoFisher Scientific). All cells were cultured at 37 °C with 5% CO_2_. TMD8‐pCasper cells (clone K4.23) were generated using the two‐component Sleeping Beauty system. The plasmid IVTRup‐SB100x was used for in vitro transcription of SB100X‐mRNA as described in.^[^ ^47^, ^48^ ^]^ pCasper Minicircle (pCasper coding sequence from Evrogen (#FP971), EF1A promoter, ITR (from VectorBuilder) was generated by PlasmidFactory GmbH. SB100X‐mRNA and pCasper Minicircle (ratio 5:1) were nucleofected using Nucleofector 4D technology (SF kit, pulse code EW‐113). TMD8 populations were sorted (Cell Sorter SH800S (Sony)) followed by single‐cell cloning. Generation of minicircles was supported by the IRTG/SFB1027 mini‐proposal 2024 for Joanne Vialle.

### Generation of CD19‐CAR‐NK Cells

CD19‐CAR‐NK cells were generated with the Sleeping‐Beauty transposon system as previously described.^[^ ^49^, ^50^ ^]^ Briefly, primary human NK cells were isolated from buffy coats obtained from healthy donors (DRK‐Blutspendedienst Baden‐Württemberg‐Hessen) and were cultivated in NK‐MACS (Miltenyi Biotec) supplemented with 1% NK‐MACS Supplements (Miltenyi Biotec), 1% Penicillin‐Streptavidin (ThermoFisher Scientific), 5% heat‐inactivated human plasma (DRK‐Blutspendedienst Baden‐Württemberg‐Hessen), and 120 U mL^−1^ IL‐15 (Miltenyi Biotec). For CD19‐CAR‐NK cell generation, 1 × 10^6^ NK cells were nucleofected with 1.0 µg CD19‐CAR minicircle DNA (PlasmidFactory) and 2.5 µg SB100X transposase mRNA (Ethris) using the DO100 program, the P3 Primary Cell 4D‐Nucleofector X Kit S (Lonza), and 4D Nucleofector (Lonza).

### Cryopreservation of NK Cells

CD19‐CAR‐NK cells were cryopreserved at a density of 5 × 10⁶ cells mL^−1^ between 14 and 21 days after nucleofection using RPMI medium (ThermoFisher Scientific) supplemented with 20% FBS and 10% DMSO (AppliChem) and 1% Penicillin‐Streptavidin. Freshly isolated NK cells were resuspended in freezing medium (90% FCS, 10% DMSO) at a density of 6 × 10⁶ cells mL^−1^. For thawing, cryovials were rapidly warmed in a 37 °C water bath with gentle agitation for < 2 min until ice crystals fully dissolved. Cells were immediately centrifuged (300 × g, 5 min) to remove cryoprotectant solution, then resuspended in pre‐equilibrated AIM V medium supplemented with 10% FCS. For revitalization, thawed NK or CAR‐NK cells were co‐cultured with bead‐activated CD4⁺ T cells or synthetic cells under standard conditions (37 °C, 5% CO_2_) for 24 h.

### Real Time Killing Assay for 2D Settings

Target cells were loaded with calcein‐AM (500 nM) for 15 min at RT and then plated onto a Falcon 96‐well Black/Clear Flat Bottom TC‐treated Imaging Microplate (Corning) at a density of 2.5 × 10^4^ cells well^−1^. For ADCC, rparaituximab (1 µg mL^−1^) was present in the media during the killing assay. Effector cells (NK cells or PBMCs) were subsequently added at the ratio indicated in the figure legends. The fluorescence was measured at 37 °C every 10 min for 4 h using either a GENiosPro micro‐plate reader (TECAN) ^[^ ^21^ ^]^ or a high‐content imaging system ImageXpress (Molecular Devices) with a 20× objective.

### Live‐Cell Imaging for 3D Killing

This assay was performed as previously described.^[^ ^25^ ^]^ Briefly, target cells were resuspended in a 2 mg mL^−1^ pre‐chilled, neutralized collagen type I solution (Advanced Biomatrix) and plated onto a Falcon 96‐well Black/Clear Flat Bottom TC‐treated Imaging Microplate (Corning) with 2.5 × 10^4^ cells in 40 µL per well, followed by incubation at 37 °C with 5% CO_2_ for 40 min for collagen solidification. Effector cells were then added from above, and killing was visualized at 37 °C with 5% CO_2_ every 20 min for 40 h using a high‐content imaging system ImageXpress (Molecular Devices) with a 20× objective. Images were analyzed using ImageJ (NIH Image).

### Live‐Cell Imaging for CD3 Transfer

Glass coverslips (25 mm) were coated with fibronectin at room temperature for 30 min, rinsed, and cells were seeded immediately. Human CD4⁺ T cells activated by beads for 2 days were stained by incubating 0.5–1 × 10^6^ cells in 50 µL PBS + 0.5% BSA with 1 µL Alexa Fluor 647‐anti‐CD3 (BioLegend OKT3) for 30 min at 4 °C in the dark, then washed with PBS + 0.5% BSA. NK cells were CFSE‐labeled and mixed with the stained T cells at a 1:2 ratio (0.5 × 10^6^ NK:1.0 × 10^6^ T) in 100 µL AIM V medium, applied to the coated coverslips, and incubated for 20 min at 37 °C/5% CO_2_; nonadherent cells were removed by gentle AIM V washes. For live imaging, coverslips were mounted on the microscope stage in 1 mL AIM V at 37 °C/5% CO_2_. Time‐lapse microscopy (30–60 min, 10 s intervals) was performed on a Zeiss Cell Observer (40×) using brightfield, CFSE (Ex 488/Em 525 nm), and Cy5 (Ex 625/Em 665 nm) channels. Images were processed and analyzed in Fiji (NIH ImageJ).

### Immunocytochemistry

Coverslips (13 mm) were coated with fibronectin at RT for 30 min. After removing excess fibronectin, cells suspended in AIM V medium were settled on the coated area and kept at 37 °C with 5% CO_2_ for 20 min. Unattached cells were gently washed away with PBS. Samples were immediately fixed in freshly prepared 4% paraformaldehyde at RT for 20 min, followed by three washes with PBS (5 min each). Permeabilization was performed using PBS containing 0.3% Triton X‐100 and 5% FCS at RT for 1 h. Then cells were stained with Alexa Fluor 647 anti‐human IL‐2 antibody and Alexa Fluor 488 anti‐human CD3 antibody in PBS containing 1% BSA at RT for 2 h. After washing, cells were mounted in 20 µL of Epredia Immu‐Mount (ThermoFischer Scientific). Samples were visualized using a Cell Observer (Zeiss) with a 60× objective.

### Migration Analysis

Visualization of NK cell migration in 3D was performed as described previously.^[^ ^51^ ^]^ Briefly, NK cells were labeled with CellTrace CFSE (5 µM) at RT for 15 min, and after washing were co‐cultured with bead‐stimulated CD4^+^ T cells or cultured alone in AIM V medium with 10% FCS for 24 h. These cells were then resuspended in a 2 mg mL^−1^ pre‐chilled, neutralized collagen type I solution (Advanced Biomatrix). Cell/collagen mix was plunged into a capillary (inner diameter: 1.5 mm, outer diameter: 2 mm) and kept at 37 °C with 5% CO_2_ for 40 min for solidification, followed by a 1‐h recovery in AIM V medium. NK cell migration was imaged at 37 °C with 5% CO_2_ every 30 s for 1 h using a Z.1 light‐sheet microscope (Zeiss) with a 20× objective. The Z‐step size was 1 µm, and in total 201 slices were obtained per stack. Cell trajectories were automatically tracked and analyzed using Imaris 8.1.2 (Bitplane). NK cells with a track duration of less than 30 min were excluded from the analysis.

To calculate the turning angle (**φ**), three successive positions of the same NK cell (with x, y, and z coordinates obtained from Imaris) were used. A φ value close to zero indicates a tendency to maintain the previous direction of movement, signifying persistent motion, whereas φ values near π represent a reversal in direction. Based on this, instantaneous persistence was defined as *cos(φ)* and the average persistence as 〈*cos(φ)*〉.

The mean square displacement (MSD) was calculated as:

(1) $$MSD\left(t\right)=\langle \langle r\left(t\right)2\rangle \rangle d\;-\langle \langle r\left(t\right)\rangle \rangle d2$$

where 〈…〉 denotes averaging over all cell trajectories within a single donor dataset, and 〈…〉_d_ represents the average over all donors. This two‐step averaging provides a robust measure of cell motility while reducing donor‐specific variability.

### Flow Cytometry Analysis of NK Cell Subsets, Proliferation, and Cytotoxic Protein Expression

NK cells were stained with Alexa Fluor 488 anti‐human CD16, APC mouse anti‐human CD56, and PerCP anti‐human CD3 antibodies at 4 °C for 30 min in the dark. NK cell proliferation was assessed by labeling freshly isolated primary human NK cells with CFSE (5 µM) at RT for 10 min before co‐culture with autologous bead‐activated CD4^+^ T cells. For perforin and granzyme B staining, cells were fixed in 4% paraformaldehyde, permeabilized with 0.1% saponin in PBS containing 0.5% BSA and 5% FCS. Data were acquired using a FACSVerse flow cytometer (BD Biosciences) and analyzed with FlowJo v10.

### RNA Sequencing and Analysis

NK cells were isolated with CD56 magnetic microbeads (Miltenyi Biotec) from NK‐T co‐culture (Boosted) or NK cultured alone (NK). Isolated NK cells (1–2 × 10⁶ per condition) were collected by centrifugation at 300 g for 10 min. After removing the supernatant, the cell pellets were immediately frozen in liquid nitrogen and stored at −80 °C until use. RNA extraction was performed simultaneously for all samples using the NucleoSpin RNA Plus kit (Macherey–Nagel), following the manufacturer's protocol. RNA concentration was measured with a NanoDrop OneC Microvolume UV–vis Spectrophotometer (ThermoFisher Scientific), with samples having an A260/A280 ratio between 2.0 and 2.2 considered pure. RNA quality was further assessed by electrophoresis on a 1% agarose gel, where samples displaying distinct 28S and 18S rRNA bands were deemed intact. RNA sequencing was conducted by Novogene using Illumina platforms based on sequencing‐by‐synthesis (SBS) technology. Gene expression levels were quantified using Fragments Per Kilobase of exon model per Million mapped fragments, which accounts for sequencing depth and gene length in fragment counting.^[^ ^52^ ^]^ Read counts obtained from gene expression analysis were utilized for differential expression analysis.

Differential gene expression between the two groups (Ctrl vs Boosted) was analyzed using the DESeq2 R package,^[^ ^53^ ^]^ which applies a negative binomial distribution model to identify statistically significant differences in gene expression. *P*‐values were adjusted using the Benjamini–Hochberg method to control the false discovery rate, with adjusted *p*‐values (padj) < 0.05 considered statistically significant for differentially expressed genes (DEGs). Functional annotation and enrichment analysis of DEGs, including Gene Ontology (GO) and Kyoto Encyclopedia of Genes and Genomes (KEGG) pathway analysis, were performed using the web‐based DAVID database.^[^ ^54^, ^55^ ^]^ For GO analysis, GO Fat categories were selected to obtain more specific functional terms.

### Synthetic Cell Preparation and Functionalization

Synthetic cells (droplet‐supported lipid bilayer, dsLB) were assembled following the strategy previously described.^[^ ^56^ ^]^ Briefly, ≈100 mg of PDMS (Sylgard 184, Dow Corning, USA) was mixed with 760 µL of PBS containing 1 mm SDS (unless stated otherwise) and manually pre‐emulsified by resuspension. The resulting mixture was transferred to a sonication bath and sonicated for 2 min at RT to generate a dispersed oil‐in‐water (o/w) emulsion. To initiate dsLB formation, MgCl_2_ was added to a final concentration of 40 mm, along with 200 µL of a 6 mm SUV solution, yielding a final lipid concentration of 600 µm. Small unilamellar vesicles (SUVs) were composed of 20 mol% EggPG, 5 mol% PE‐MBP, 1 mol% LissRhodamine B‐PE, and 74 mol% EggPC (all from Avanti Polar Lipids, USA), prepared via extrusion as previously described.^[^ ^56^ ^]^ The dsLB suspension was incubated at RT in the dark for 2 min before centrifugation at 10 000 × g for 30 s. The supernatant was discarded, and the dsLB pellet was resuspended in 1 mL of PBS with a pH adjusted to 7.0, followed by an additional centrifugation step under the same conditions. To remove remaining MgCl_2_‐SUV agglomerates and particles in a sub‐micrometer size range, the dsLBs were washed further via centrifugation at 500 × g for 1 min. The supernatant was discarded, and the particles resuspended in 1 mL of PBS (pH 7.0). This process was then repeated one more time, and the dsLBs were resuspended in 1 mL of PBS (pH 7.0) and stored at 4 °C in the dark until use.

For protein functionalization, human recombinant IL‐2 (Stemcell Technologies, USA) was added in three differing quantities to a volume of dsLB suspension (≈100 µL) corresponding to 3 × 10^6^ particles: 30, 300, and 900 IU. The specific activity of the cytokine was approximated by the supplier as 1.8 × 10^4^ IU µg^−1^. All conditions were generated in duplicates and incubated under agitation in the dark for 60 min to facilitate binding to the PE‐MBP. After incubation, dsLBs were washed via centrifugation at 10 000 × g for 30 s and resuspension in PBS (pH 7.0) equivalent to the initial volume of dsLB‐suspension. To ensure that the dsLB membrane was sufficiently saturated with the protein, it was added at a 1:1 molar ratio to the calculated accessible PE‐MBP concentration. The suspensions were incubated under agitation in the dark for 60 min and washed as described above. The functionalized dsLBs were stored at 4 °C in the dark until use. The same coating procedure was applied for human recombinant IL‐15 (Miltenyi Biotec). To boost NK cell function, NK cells were co‐cultured with synthetic cells at a 1:2 ratio for 24 h in U‐bottom plates.

### Preparation of Samples for Mass Cytometry (CyTOF)

Staining of the cells for mass cytometry analysis was performed on 1.5 × 10^6^ fresh NK cells cultured alone and 3 × 10^6^ NK cells co‐cultured with CD4^+^ T cells in a ratio 1:2. To stain the proliferating cells, cells were resuspended in 5 mL of complete RPMI media containing 10% FBS and 1%Penicillin‐Streptomycin, then 50 µM of Cell‐ID 127 IdU (Fluidigm) was added to the cell suspension and incubated for 30 min at 37 °C in 5% CO_2_. Cells were washed with PBS (without Ca^2+^/Mg^2+^) followed by centrifugation at 500 × g at RT for 5 min. Cells were then stained with 5 µm Cell‐ID cisplatin (Fluidigm) for 5 min at RT and afterward washed with PBS containing 10% FBS at 500 × g for 10 min. FC block (5 µL well^−1^) Human TruStain FcX (Biolegend) was added for 10 min at RT. For most markers, heavy‐metal labeled antibodies were commercially available and were purchased from Fluidigm. For the other markers (^*^) heavy‐metal labeling was performed using the Maxpar X8 Multimetal Labeling kit (Fluidigm) according to the manufacturer's instructions. Extracellular surface staining was performed in 96‐well plates by first adding a cocktail of pre‐conjugated and homemade‐conjugated antibodies (Table S1, Supporting Information) for 30 min at RT. The excess of antibodies was removed by washing with PBS + 10% FBS. Subsequently, a secondary antibody cocktail (Table S2, Supporting Information) was added and incubated for 30 min at RT, and remaining antibodies were removed by washing with PBS with 10% FBS. Cells were fixed and permeabilized using the Fixation/Permeabilization kit (eBioscience) according to the manufacturer's instructions. Intracellular staining was performed by adding a cocktail of pre‐conjugated antibodies (Table S3, Supporting Information) for 30 min at RT, and excess antibodies were removed by washing with PBS with 10% FBS. Cells were stained with 50 nm cell‐ID Intercalator‐Ir (Fluidigm) in Maxpar Fix & Perm buffer (Fluidigm) overnight according to the manufacturer's instructions. Prior to acquisition, fixed cells were washed twice with PBS and then twice with deionized water. Cells were resuspended at a density of 1.5 × 10^6^ cells mL^−1^ in deionized water including 10% calibration beads (EQ Four Element Calibration Beads, Fluidigm), and the samples were analyzed with the Helios mass cytometer (Fluidigm) at a flow rate of 0.030 mL min^−1^. After acquisition, fcs files were normalized (CyTOF software version 6.7, Normalization Passport EQ‐P13H2302_ver2) by using EQ four‐element calibration beads (Fluidigm, ref. 201 078) and randomized.

### Mass Cytometry Data Analysis

Using FlowJo software, samples were pre‐gated for cells, including proliferating and non‐proliferating (Idu ±) cells, singlets, and live cells. Respective. fcs files were exported, and then the CyTOF workflow based on the R/Bioconductor packages “flowCore” and “CATALYST” was used for advanced analysis. “diffcyt” was used for the differential analysis of abundance, and “ggplot2” was used for visualization.

### Statistical Analysis

Data were presented as mean ± SEM unless otherwise mentioned. Statistical analysis was performed using Excel (Microsoft) or GraphPad Prism 6 (GraphPad Software). Statistical comparisons were conducted as indicated in the figure legends: Mann–Whitney U‐test or paired *t*‐test for two‐group comparisons, one‐way ANOVA with Tukey's multiple comparisons for multiple‐group analyses, and two‐way ANOVA with multiple comparisons for curve analyses. For experiments with small sample sizes (n ≤ 4), ANOVA was conducted in an exploratory manner with a reasonable assumption of normality, as the data were generated from primary cells isolated from randomly selected healthy donors. Significance levels were indicated as follows: ^*^ *p* < 0.05, ^**^ *p* < 0.01, ^***^ *p* < 0.001; ns, not significant.

### Ethical Approval

This research was approved by the local ethics committee (Approval No. 84/15; Prof. Dr. Rettig‐Stürmer). Leukocyte reduction system chambers, a by‐product of platelet collection from healthy blood donors, were obtained from the local blood bank at the Institute of Clinical Hemostaseology and Transfusion Medicine, Saarland University Medical Center. All donors provided written informed consent for the use of their blood for research purposes. The CD19‐CAR‐NK related study was approved by the Ethics Committee of the Goethe University (Approval No. 329/10).

## Conflict of Interest

E.U. has a sponsored reserach project with Gilead and BMS and acts as a medical advisor for Phialogics and CRIION.

## Author Contributions

X.Z. performed most of the experiments and the corresponding analysis and made most of the figures if not mentioned otherwise; S.Z. performed experiments for Figure 7 and Figure S9 (Supporting Information), and helped making Figures 3i and 6d–f, Figure S6c (Supporting Information), and the graphical abstract; W.Y. performed experiments for Figure S3 (Supporting Information); S.G. and A.L. performed CyTOF experiments and initial analysis; J.P. and E.M. designed CyTOF experiments and performed further analysis; E.M. provided Figure 2, Figures S4 and S5 (Supporting Information); Z.S. and H.R. analyzed NK migration trajectories and provided Figure 3c,d; N.P. and O.S. provided synthetic cells and Figure 6c; A.M. and E.U. provided cryopreserved CAR‐NK cells and Figure S10 (Supporting Information); S.S. helped with flow cytometry; N.K. and L.K. helped with expanding CAR‐NK cells; G.S. and E.C.S. provided TMD8‐pCasper cells; H.E. provided LRS‐chambers for PBMC preparation; M.H. helped with data interpretation and provided critical feedback on all aspects of the project; B.Q. generated concepts, designed experiments, and wrote the manuscript; All authors contributed to the writing, editing, and cross‐checking of the manuscript.

## Supporting information



Supporting Information

Supplemental Movie 1

Supplemental Movie 2

Supplemental Movie 3

Supplemental Movie 4

Supplemental Movie 5

Supplemental Movie 6

## Data Availability

The datasets generated and analyzed during the current study are available from the corresponding author upon reasonable request.
