# Supplementary material for: Restoring NK Cell Cytotoxicity Post‐Cryopreservation via Synthetic Cells
Source: Adv Sci (Weinh). 2025 Sep 18;12(42):e05731. doi: 10.1002/advs.202505731 (PMC12622558; doi:10.1002/advs.202505731)
Supplement: Supplementary file 1 — Supporting Information [file ADVS-12-e05731-s007.pdf]

Restoring NK Cell Cytotoxicity Post-Cryopreservation via Synthetic Cells

Xiangda Zhou[1,2], Sijia Zhang[1], Wenjuan Yang[1#], Susanne Gonder[3], Zeinab Sadjadi[4], Nils Piernitzki[5], Alina Moter[6,7], Shulagna Sharma[1], Anne Largeot[3], Nadja Küchler[1], Lea Kaschek[1], Gertrud Schäfer[1], Eva C. Schwarz[1], Hermann Eichler[9], Evelyn Ullrich[6,7,8], Heiko Rieger[4], Oskar Staufer[5], Jérôme Paggetti[3], Etienne Moussay[3], Markus Hoth[1], Bin Qu[1,10]

Supplementary Materials

Supplementary Method

Multiplex cytokine assay

Supernatant samples were collected using two-step centrifugation process: 1) 300g for 10 min, followed by 2) 1000g for 5 min. Aliquots (150 µl per tube) were stored at -80°C until use. IL-2 levels were quantified using the LEGENDplex Human CD8/NK Panel (BioLegend) with a 96-well V-bottom Plate (BioLegend), following the manufacturer's instructions. Data acquisition was carried out with a flow cytometer equipped with an autosampler (BD Biosciences), collecting 4000 events per sample. Data analysis was conducted using the LEGENDplex Data Analysis Software (BioLegend).

Supplementary figures and legends

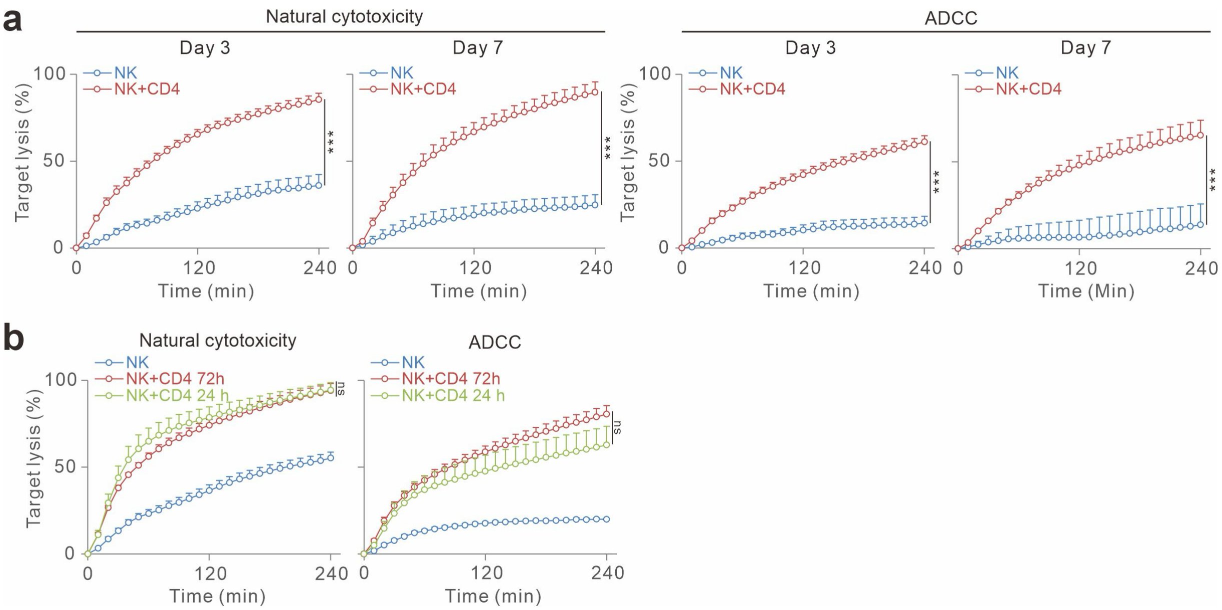

Supplementary Figure 1. Duration of T cell co-culture required for enhancing NK cytotoxicity. NK cell killing dynamics was determined using the plate-reader based 2D real-time killing assay. K562 (natural cytotoxicity) and Raji (ADCC) cells were used as target cells (E:T = 2.5:1). (**a**) NK cells were co-cultured with $CD4^+$ T cells in presence of beads for 3 or 7 days. The killing assay was performed on Day 3 or Day 7 as indicated. Results for 3 days are the same as Fig. 1b. (**b**) NK cells were co-cultured with bead-stimulated $CD4^+$ T cells on Day 2 post-isolation for 24 hours and then used for the killing assay. Results are shown as mean ± SEM (n = 4 donors).

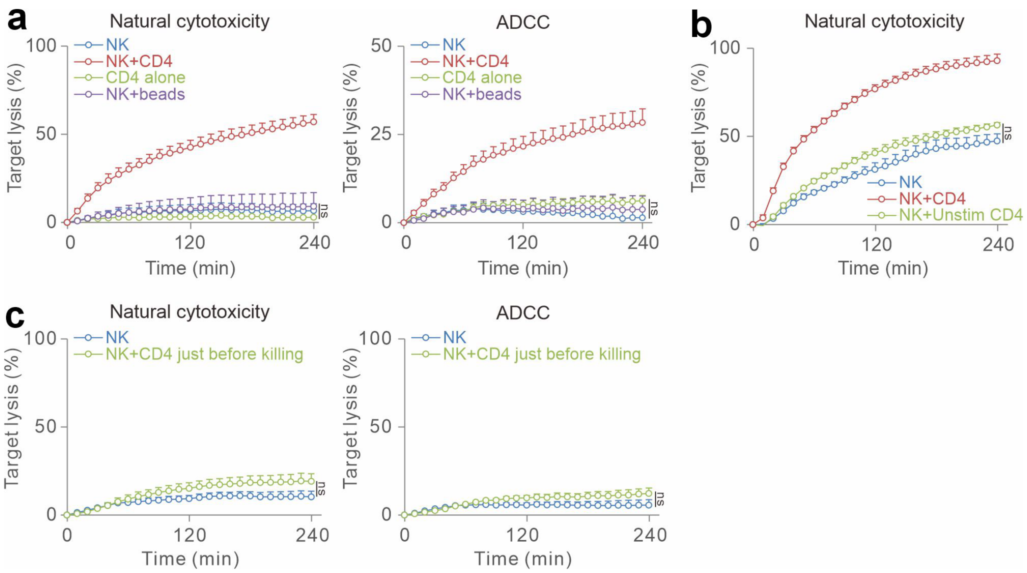

Supplementary Figure 2. Effector T cells are required to enhance NK cell cytotoxicity. NK cell killing dynamics was determined using the plate-reader based 2D real-time killing assay. K562 (natural cytotoxicity) and Raji (ADCC) cells were used as target cells (E:T = 2.5:1). (**a**,**b**) NK cells were cultured alone (NK) or co-cultured with bead-stimulated CD4$^+$ T cells (NK+CD4), with CD3/CD28 beads (NK+beads), or with unstimulated CD4$^+$ T cells (NK+Unstim CD4) for 3 days. Bead-stimulated CD4$^+$ T cells were also used as effector cells (CD4 alone). (**c**) Bead-activated T cells were mixed with NK cells right before the start of the killing assay. Results are shown as mean ± SEM (n = 4 donors).

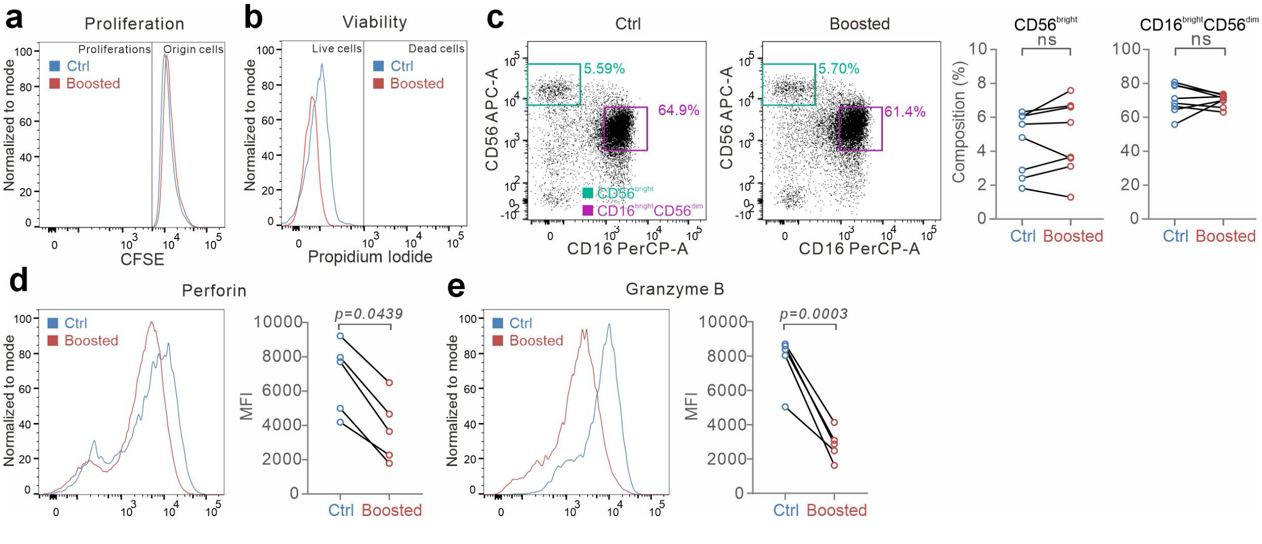

Supplementary Figure 3. NK proliferation, viability, subpopulations and degranulation remain unchanged with T cell co-culture. NK cells were either cultured alone (Ctrl) or co-cultured with bead-activated T cells (Boosted) for 3 days. (**a-c**) CD3, CD56 and CD16 were stained to distinguish NK cells from T cells. For proliferation (**a**), NK cells were pre-loaded with CFSE. For viability (**b**), NK cells co-cultured with T cells were stained with propidium iodide. NK subpopulations CD56$^+$CD16^{hi} and CD56$^+$CD16^{dim} were examined in **c.** Results are from 8 donors. (**d**, **e**) Expression of perforin and granzyme B. Cells were fixed and stained with CD3, CD56 and CD16 along with perforin (**d**) or granzyme B (**e**). Protein expression was analyzed using flow cytometry. Results are from 5 donors. Paired t-test was performed for statistical analysis for **c-e**.

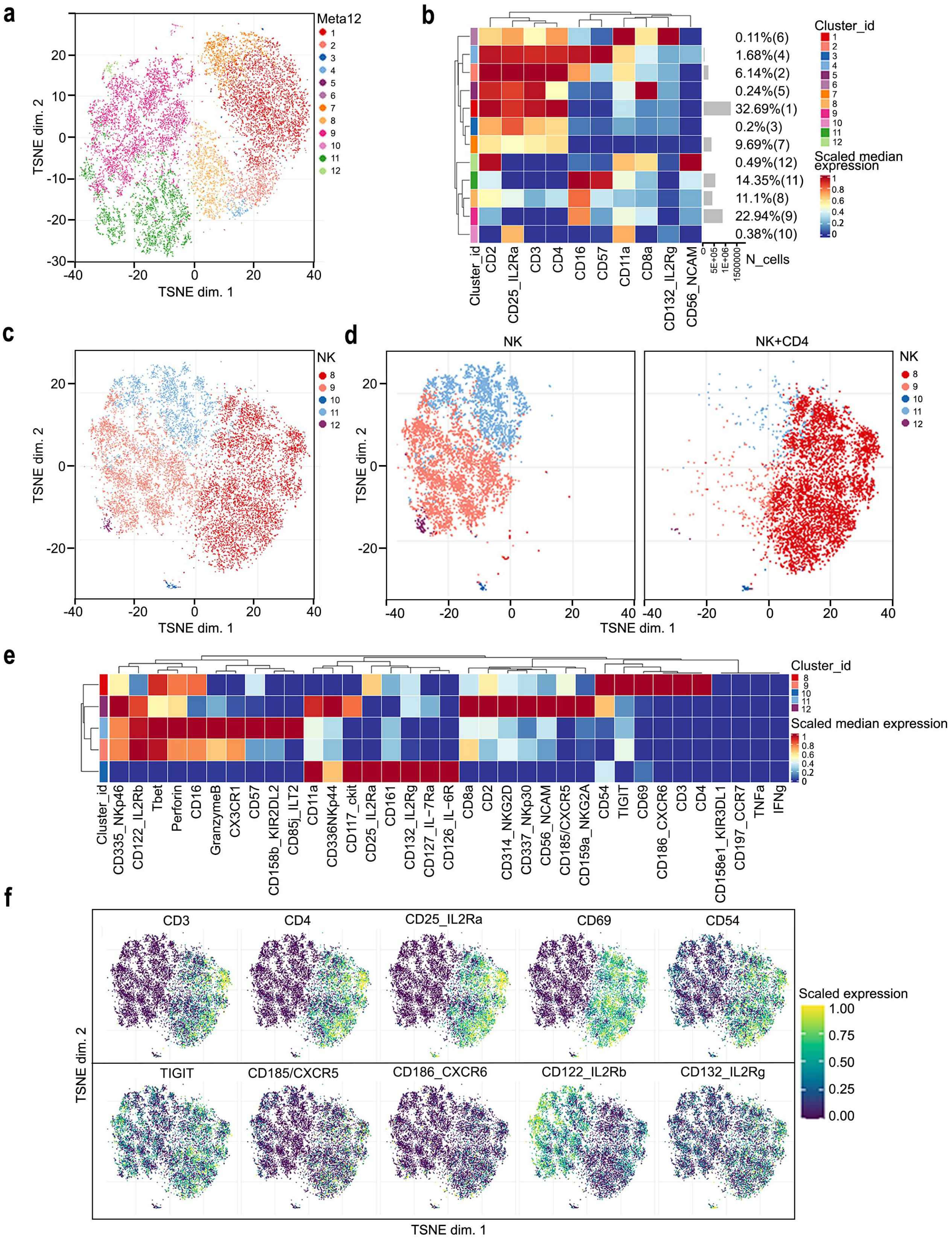

Supplementary Figure 4. Characterization of NK cell subsets using CyTOF. Primary NK and CD4^{+} T cells were isolated from six donors. NK cells were either cultured alone (NK) or co-cultured with autologous bead-stimulated CD4^{+} T cells for 3 days (NK+CD4). (**a**) t-SNE plot depicting 12 metaclusters of cells. (**b**)

Expression of lineage and subset markers used for identifying distinct cell types and subsets. (**c**) t-SNE plot highlighting five NK cell clusters (clusters 8–12). (**d**) t-SNE plot showing NK cell distribution under different conditions (NK vs. NK+ CD4). (**e**) Heatmap displaying marker expression across the five NK cell clusters. (**f**) t-SNE plots of selected markers illustrating changes in marker expression.

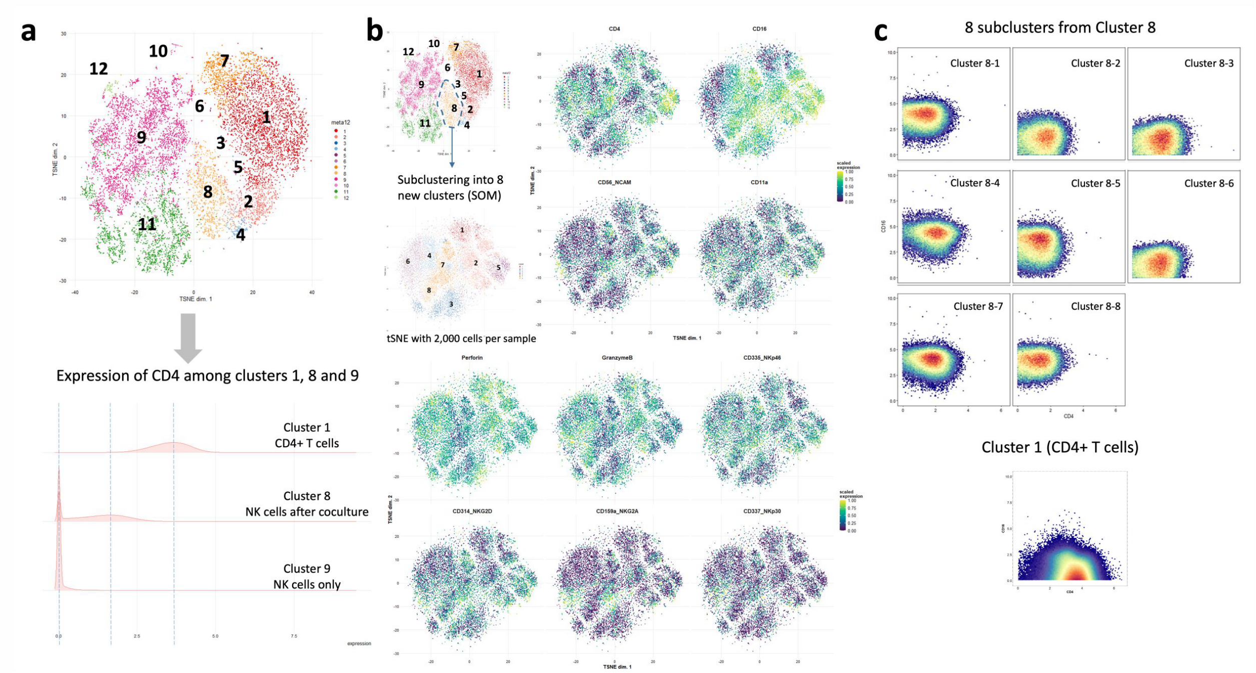

Supplementary Figure 5. Phenotype and CD4 expression of NK cells following co-culture with T cells. (**a**) T-SNE plot of the 12 metaclusters of cells (upper panel) and CD4 expression in cells of Cluster 1 (CD4+ T cells), Cluster 8 (NK after co-culture), and Cluster 9 (NK cells without co-culture). (**b**) T-SNE plots of the initial clustering and newly generated clusters form cluster 8 performed with FlowSOM, showing the new 8 subclusters (8-1 to 8-8) and the expression of CD4 and NK cell markers. (**c**) Scatter plots showing the expression of CD4 in the 8 subclusters from Cluster 8 and in the initial Cluster 1 ($CD4^{+}$ T cells).

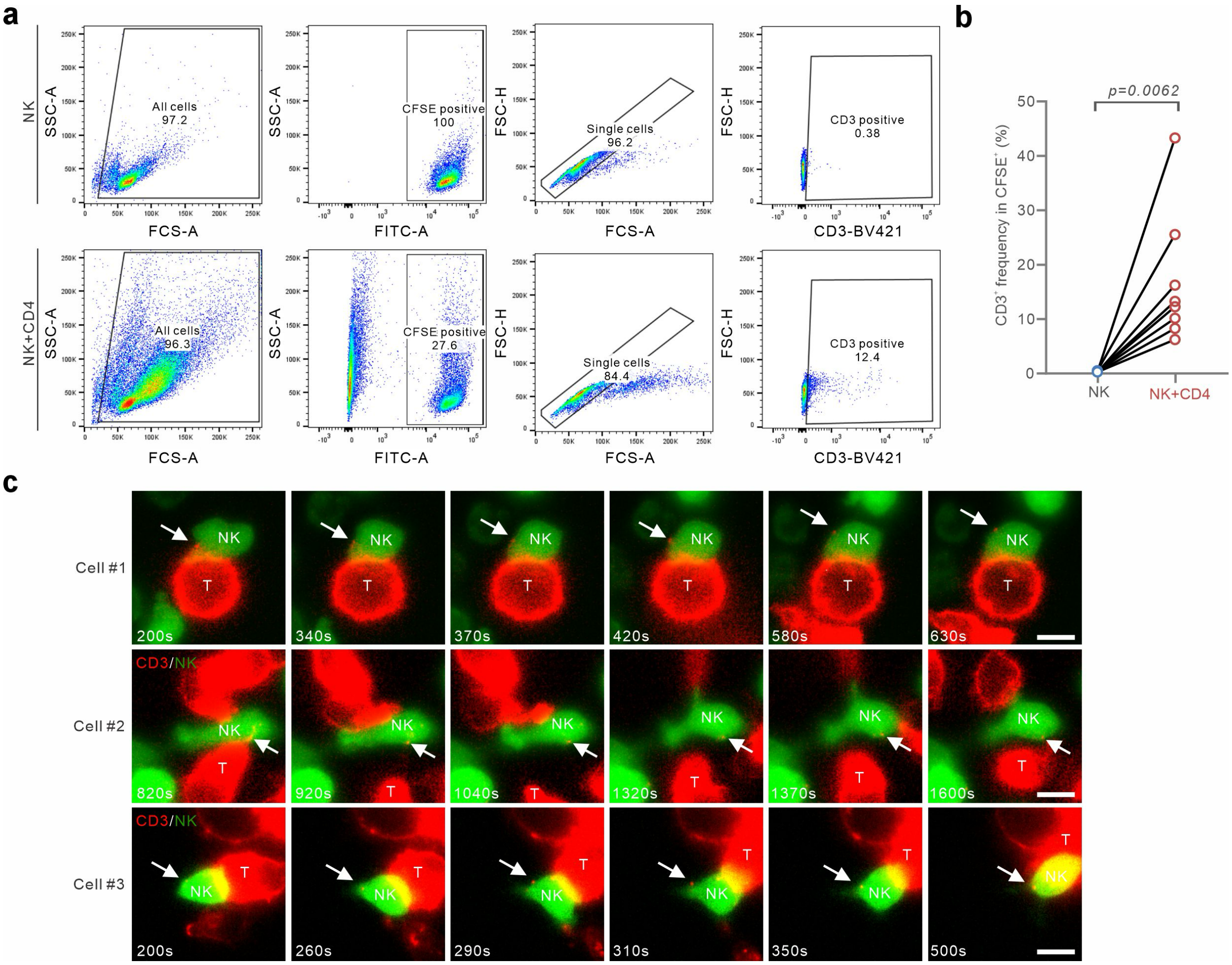

Supplementary Figure 6. CD3 can be transferred from T cells to NK cells upon contact. CFSE-labeled NK cells were co-cultured with bead-stimulated $CD4^+$ T cells pre-stained with anti-CD3 antibody. Samples were analyzed using flow cytometry (**a-b**) or live-cell imaging (**c**). (**a-b**) Identification of $CD3^+$ NK cell fraction after T cell-co-culture. The gating strategy is shown. MFI: mean fluorescence intensity. One representative donor is shown in **a**. Quantification is shown in **b** with paired t-test for statistical analysis. Results were from 8 donors. (**c**) Live cell imaging shows transfer of CD3 from T cells to conjugated NK cells. Three representative cells are shown. Transferred CD3 dots are highlighted with white arrowheads. Time-lapse imaging was performed using a Cell Observer microscope (Zeiss) at 37 °C with 5% CO_2, with images acquired every 10 seconds. Channels showed: green fluorescence (Ex 488/Em 525 nm for CFSE), and Cy5 (Ex 625/Em 665 nm for Alexa Fluor 647). Scale bars = 20 μm.

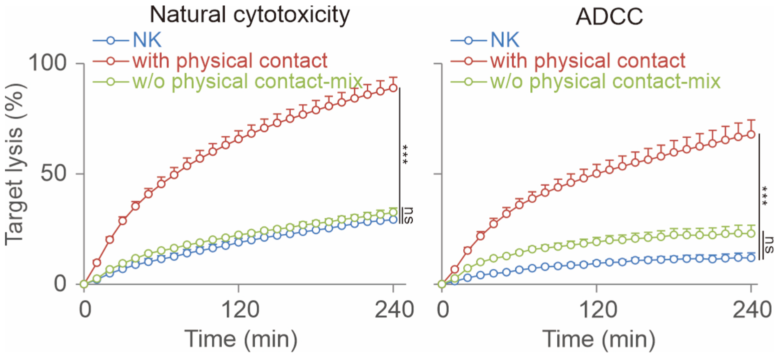

Supplementary Figure 7. Conditional medium fails to rescue NK cytotoxicity. Conditional medium of outer wells (related to Fig. 5a) was manually transferred to the insert to ensure accessibility of NK cells to T cell-derived soluble factors (w/o physical contact-mix). NK cell killing kinetics was determined using the plate reader-based 2D real-time killing assay (E:T ratio = 2.5:1, n = 4 donors). Statistical analysis was conducted via a two-way ANOVA with multiple comparisons.

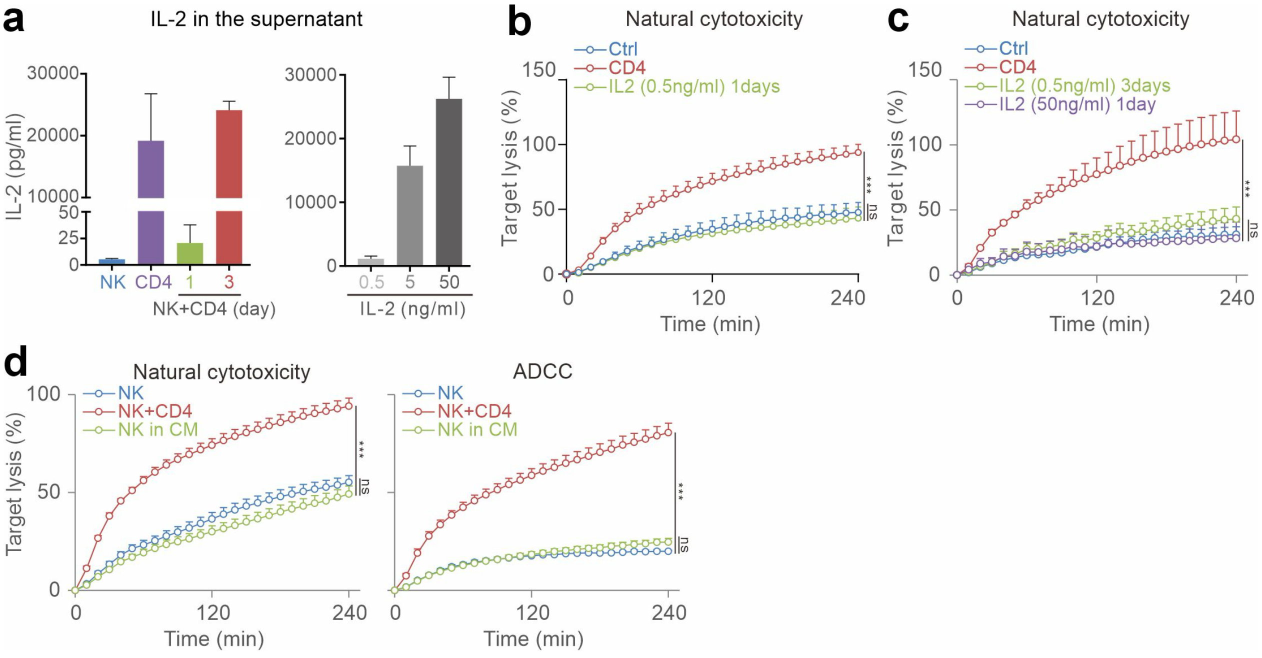

Supplementary Figure 8. Global IL-2 fails to enhance NK cell cytotoxicity to a comparable level as T cell co-culture. (**a**) IL-2 concentrations in supernatants were quantified using a multiplex cytokine assay. Supernatants were taken on Day 1 and Day 3 from NK-CD4 co-culture, or on Day 3 from $CD4^+$ T cells alone (CD4) and NK cells alone (NK). Recombinant human IL-2 was added to NK cell culture, and the supernatant was yielded for analysis on Day 3. Results are shown as mean ± SD (n = 4 donors). (**b**,**c**) NK cells were cultured in the presence of IL-2 for 1 or 3 days. NK cell killing kinetics was determined by the plate reader-based 2D real-time killing assay (E:T ratio = 2.5:1, mean ± SEM, n = 4 donors). (**d**) NK cells were cultured alone (NK), with bead-activated $CD4^+$ T cells (NK+CD4) or with $CD4^+$ T cell conditional medium (NK in CM). NK cell killing kinetics was determined by the plate reader-based 2D real-time killing assay (E:T ratio = 2.5:1, mean ± SEM, n = 4 donors). Statistical analysis was conducted via a two-way ANOVA with multiple comparisons for **b**-**d**.

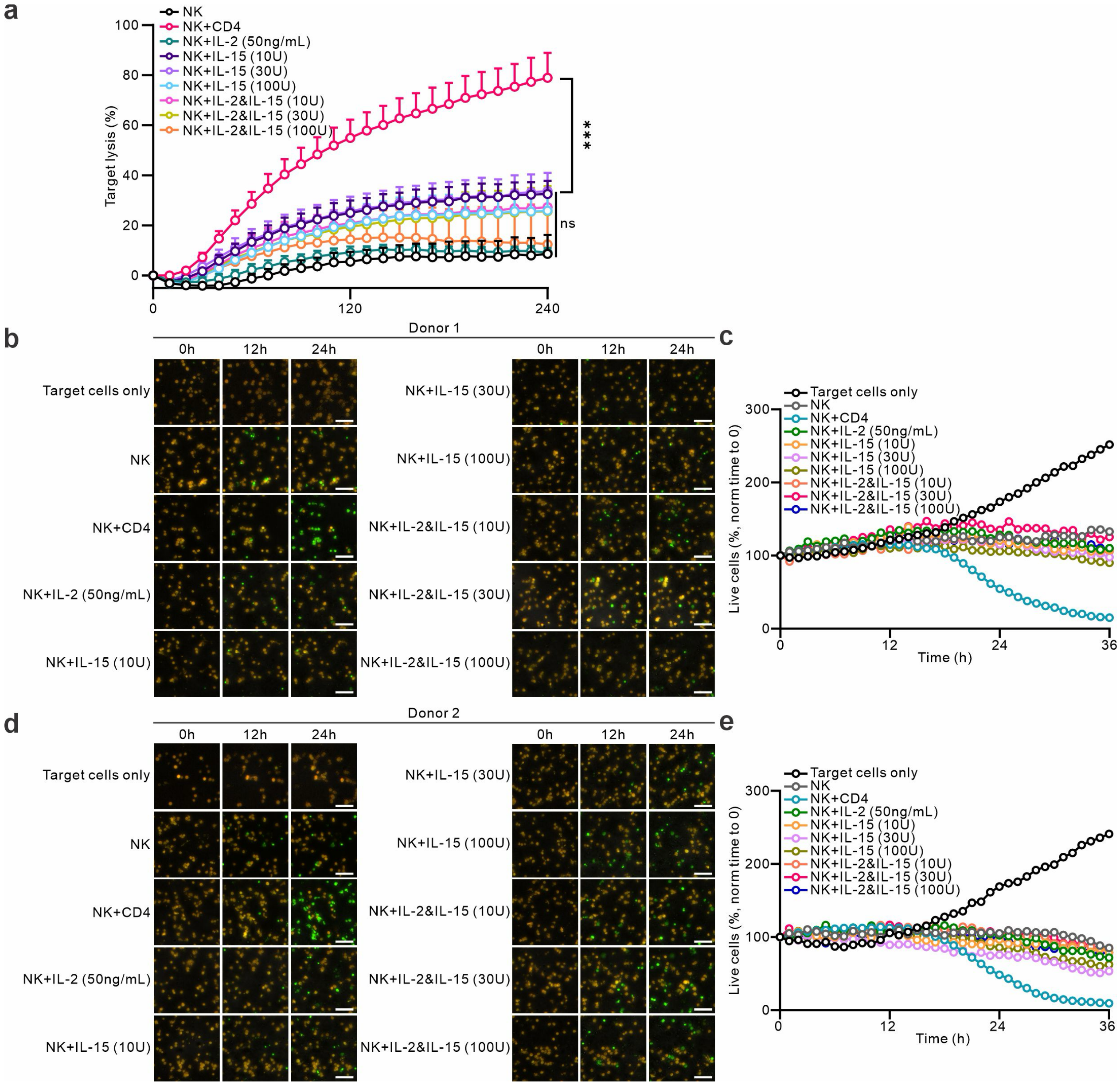

Supplementary Figure 9. Soluble IL-15 slightly enhances NK cell cytotoxicity. NK cells were co-cultured for 24 hours with either soluble cytokines (IL-2, IL-15, or their combination) or autologous bead-activated $CD4^+$ T cells. (**a**) NK cell cytotoxicity was analyzed using plate reader-based 2D real-time killing assay with K562 cells as targets (E:T ratio = 2.5:1). Data are presented as mean ± SEM from 4 donors. Statistical analysis was performed using two-way ANOVA with multiple comparisons. (**b**-**e**) NK cell cytotoxicity was evaluated using a 3D real-time killing assay using high-content imaging (ImageXpress, 20× objective). K562-pCasper target cells were used as targets with an E:T ratio of 2.5:1. Time lapse from two donors are shown in **b** and **d**, the corresponding quantification for each time point is shown in **c** and **e**. Scale bars = 40 μm.

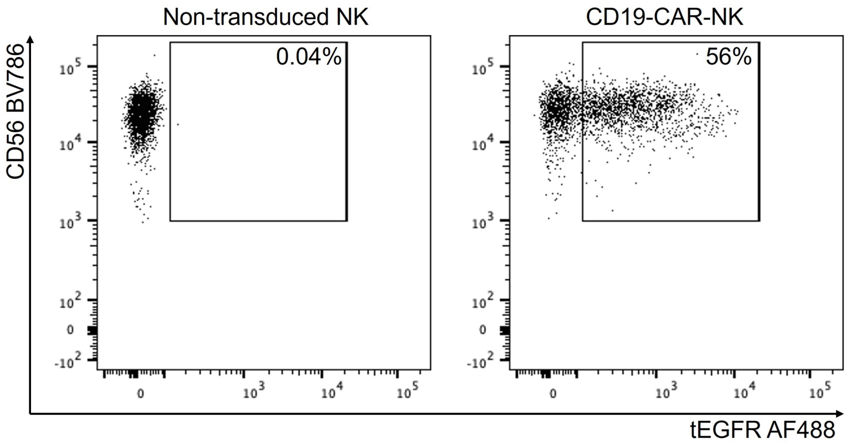

Supplementary Figure 10. Expression of CD19-targeting CAR on transduced NK cells. Representative flow cytometry-based dot plots from one donor of CD19-CAR-NK and non-transduced NK cells expressing tEGFR on CD56+NK cells (gated on CD56+CD3- NK cells). The tEGFR is co-expressed with CD19-CAR on NK cells, functioning as a safety mechanism and detection of CAR+ NK cells.

Supplementary movie legends

Movie 1 (related to Fig.1d): NK cell-mediated natural cytotoxicity in 2D settings assessed by live-cell imaging. K562 cells were loaded with calcein and seeded with NK cells (E:T = 2.5:1). Killing events were visualized every 10 min for 4 hours at 37°C using high-content imaging (ImageXpress, 20× objective). Scale bars: 40 µm.

Movie 2 (related to Fig. 1e): NK cell-mediated ADCC in 2D settings assessed by live-cell imaging. K562 cells were loaded with calcein and seeded with NK cells (E:T = 2.5:1). Killing events were visualized every 10 min for 4 hours at 37°C using high-content imaging (ImageXpress, 20× objective). Scale bars: 40 µm.

Movie 3 (related to Fig. 1f): Killing dynamics of isolated NK cells from NK-T co-culture. NK cells were isolated with CD56 magnetic beads from NK-T co-culture (Iso NK) or NK cultured alone (NK). Cytotoxicity against K562-pCasper cells (E:T = 2.5:1) was assessed via live-cell imaging every 10 min for 4 hours at 37°C using high-content imaging (ImageXpress, 20× objective). Scale bars: 40 µm.

Movie 4 (related to Fig. 3a): NK cell migration in 3D environments. Primary NK cells were either cultured alone for three days (NK) or co-cultured with autologous bead-stimulated CD4+ T cells on Day 2 post-isolation for 24 hours (NK+CD4). NK cells were loaded with CFSE on Day 1 post-isolation. For live-cell imaging, cells were embedded in a collagen matrix (2 mg/mL) and NK cell movements were visualized via light-sheet microscopy (20× objective) every 30 sec for 60 min at 37°C.

Movie 5 (related to Fig. 4b): NK cells were isolated with CD56 magnetic beads from NK-T co-culture (Iso NK) or NK cultured alone (NK). (b) Killing efficiency against K562-pCasper target cells (E:T = 2.5:1) was determined via a 3D real-time killing assay using a high-content imaging system (ImageXpress, 20× objective). Scale bars: 40 µm.

Movie 6 (related to Fig. 6d, Donor 2): Synthetic IL-2 presenting T cells rescue NK cell killing efficiency. NK cells were co-cultured for 24 hours with synthetic cells (SynT) with surface bound IL-2 (30U, 300U, 900U) or with autologous bead-stimulated $CD4^+$ T cells. NK cells killing kinetics was assessed using the 3D real-time assay (E:T = 2:1) and visualized via high-content imaging (ImageXpress, 20× objective). Scale bars: 40 µm.

Supplementary tables and legends

Table S1. Antibody panel for surface molecules used for CyTOF

Isotope	Fluorochrome/Molecule	Epitope	Clone	Reference
**141Pr***		CD3	UCHT1	300402
**142Nd**		CD11a	HI111	3142006B
**143Nd**		CD117 (ckit)	104D2	3143001B
**144Nd**		CD69	FN50	3144018B
**145Nd***		CD4	RPA-T4	300502
**146Nd***		CD8a	RPA-T8	301002
**148Nd**		CD132	TUGh4	3148014B
**149Sm**		CD25 (IL-2R)	2A3	3149010B
	Biotin	CD126 (IL-6R)		
**151Eu**		CD2	TS1/8	3151003B
**153Eu**		CD185/CXCR5	RF8B2	3153020B
**154Sm**		TIGIT	MBSA43	3154016B
**155Gd**		CD56 (NCAM)	B159	3155008B
**156Gd**		CD85j (ILT2)	GHI/75	3156020B
**159Tb**		CD337 (NKp30)	Z25	3159017B
**160Gd**		CXCR6/CD186	K041E5	3160016B
**162DY**		CD335 (NKp46)	BAB281	3162021B
	APC	CD336 NKp44		
**164Dy**		CD161	HP-3G10	3164009B
	PE	CD197 (CCR7)		
**166Er**		CD314 (NKG2D)	ON72	3166016B
**167Er**		CD158e1 (KIR3DL1, NKB1)	DX9	3167013B
**168Er**		CD127 (IL-7Ra)	A019D5	3168017B
**169Tm**		CD159a (NKG2A)	Z199	3169013B
**170Er**		CD122	Tu27	3170004B
**172Yb**		CX3CR1	2A9-1	3172017B
**173Yb**		CD158b (KIR2DL2/L3, NKAT2)	DX271	3173010B
	FITC	CD54		
**176Yb**		CD57	HCD57	3176019B
**209Bi**		CD16	3G8	3209002B

Isotope-conjugated antibodies were purchased from Fluidigm. Isotopes marked with * were conjugated in-house to respective purified monoclonal IgG antibodies (supplier and ref see table) with the Maxpar X8 Multimetal Labeling kit (Fluidigm, ref. 201300) according to the manufacturer’s instructions.

Table S2. Secondary antibody cocktail for CyTOF

Isotope	Fluorochrome	Epitope	Clone	Reference
**163Dy**		APC	APC003	3163001B
**165Ho**		PE	PE001	3165015B
**150Nd**		Biotin	1D4-C5	3150008B
**174Yb**		FITC	FIT22	3174006B

Isotope-conjugated antibodies were purchased from Fluidigm.

Table S3. CyTOF antibody panel for intracelluar molecules

Isotope	Fluorochrome	Epitope	Clone	Reference
**152Sm**		TNFa	Mab11	3152002B
**158Gd**		IFNg	B27	3158017B
**161Dy**		Tbet	4B10	3161014B
**171Yb**		Granzyme B	GB11	3171002B
**175Lu**		Perforin	B-D48	3175004B

Antibodies were purchased from Fluidigm.
